# [1,3]Thiazolo[3,2-b][1,2,4]triazolium Salts as Effective Antimicrobial Agents: Synthesis, Biological Activity Evaluation, and Molecular Docking Studies

**DOI:** 10.3390/ijms26146845

**Published:** 2025-07-16

**Authors:** Mykhailo Slivka, Boris Sharga, Daryna Pylypiv, Hanna Aleksyk, Nataliya Korol, Maksym Fizer, Olena I. Fedurcya, Oleksandr G. Pshenychnyi, Ruslan Mariychuk

**Affiliations:** 1 Educational Scientific Institute of Chemistry and Ecology, Uzhhorod National University, Fedyntsa Str. 53/1, 88000 Uzhhorod, Ukraine; mikhailo.slivka@uzhnu.edu.ua (M.S.); hanna.hryhorka@uzhnu.edu.ua (H.A.); nataliya.korol@uzhnu.edu.ua (N.K.); 2 Faculty of Biology, Uzhhorod National University, Voloshyna Str. 32, 88000 Uzhhorod, Ukraine; boris.sharga@uzhnu.edu.ua; 3 Faculty of Medicine, Uzhhorod National University, Narodna Sq. 1, 88000 Uzhhorod, Ukraine; mf.pylypiv.daryna@student.uzhnu.edu.ua; 4 Department of Chemistry, University of Nevada, Reno, 1664 N. Virginia Street, Reno, NV 89557-0216, USA; mmfizer@gmail.com; 5 Transcarpathian Regional Center for Disease Control and Prevention of the Ministry of Health of Ukraine, Sobranetska Str. 96, 88000 Uzhhorod, Ukraine; fed.leno4ka@gmail.com (O.I.F.); agp0311cec@gmail.com (O.G.P.); 6 Department of Ecology, Faculty of Humanity and Natural Sciences, University of Presov in Presov, 17th November Str. 1, 08001 Presov, Slovakia

**Keywords:** antibacterial and antifungal action, thiazole, triazole, electrophilic heterocyclization, DFT, molecular docking

## Abstract

This study focuses on the search for new effective synthetic antimicrobial compounds as a tool against the widespread presence of microorganisms resistant to existing drugs. Five derivatives of [1,3]thiazolo[3,2-b][1,2,4]triazoles were synthesized using an accessible protocol based on electrophilic heterocyclization and were characterized using infrared (FTIR) and nuclear magnetic resonance (NMR) spectroscopies, and their in vitro antimicrobial and antifungal activities were evaluated using the agar plate diffusion method and the microdilution plate procedure. Both antibacterial (Gram-positive and Gram-negative) and antifungal activities were found for the examined samples. The minimum inhibitory concentration (MIC) varied from 0.97 to 250 µg/mL, and the minimum bactericidal concentration (MBC) from 1.95 to 500 µg/mL. Compound **2a** showed good antifungal action against *Candida albicans* and *Saccharomyces cerevisiae* with minimum fungicidal concentration (MFC) 125 and MIC 31.25 µg/mL. The molecular docking revealed that the 2-heptyl-3-phenyl-6,6-trimethyl-5,6-dihydro-3H-[1,3]thiazolo[3,2-b][1,2,4]triazol-7-ium cation stands out as a highly promising candidate for further investigation due to a wide range of interactions, including conventional hydrogen bonds, π–σ, π–π T-shaped, and hydrophobic alkyl interactions. The synthesis and preliminary evaluation of [1,3]thiazolo[3,2-b][1,2,4]triazoles yielded promising antimicrobial and antifungal candidates. The diverse interaction profile of the 2-heptyl derivative salt allows this compound’s selection for further biological studies.

## 1. Introduction

One of the priority areas for the development of science and technology is the search for new high-performance functional materials for the needs of medicine. This need has grown even more in connection with modern global challenges, like the multiple drug resistance of disease agents [1,2], and faces new challenges, such as long-term and large-scale military conflicts like the war in Ukraine [3]. Thus, searching for new synthetic antimicrobial agents may help in the solution of the drug resistance problem. Particularly, new biologically active compounds of heterocyclic nature play an important role in this regard [4,5,6,7]. Derivatives of 1,3-thiazole and 1,2,4-triazole are actively studied as potential bactericidal agents [8,9]. Over the past five years, the number of Scopus publications on the biological activity of compounds containing triazole and thiazole fragments is more than 10,000! This attracts our keen interest in condensed heterocyclic systems that combine these frameworks [10,11]. It should be noted that in addition to the search for new fused heterocycles with maximum biological effect, researchers pay special attention to the possibility of using simple-to-implement, energy-resource/conserving technologies that operate with low toxicity and affordable reagents. For this purpose, technologies of multicomponent synthesis [12,13,14], electrophilic cyclization [15], catalytic highly selective processes [16], and microwave irradiation [12,14,17,18], as well as methods that operate with non-toxic or low-toxic solvents or solvent-free reactions [18,19], are often used. All these approaches and techniques have helped to evaluate the significance of obtained compounds and to design further transformations with the aim of increasing their biological activity.

**Figure 1:**
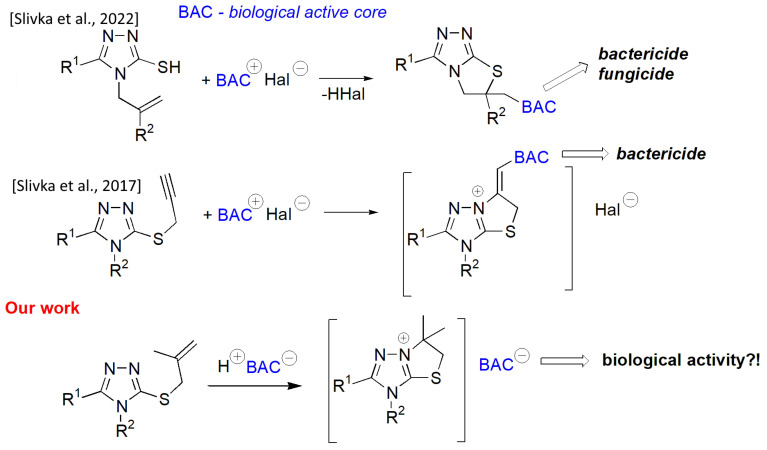
Synthetic approaches to thiazolo[1,2,4]triazoles: according to Slivka et al., 2017 [20], Slivka et al., 2022 [21], and this study.

In our previous studies, we obtained fused 1,2,4-triazoles [20,21,22] via a low-cost and efficient electrophilic cyclization methodology [15] that also allows for introducing pharmacophore fragments of various natures into the target molecule. We have used biologically active core (BAC) as an electrophile in these syntheses, which allows us to introduce BAC into a fused heterocyclic system and receive bioactive samples (Figure 1).

We previously investigated [22] the chemical properties of the [1,3]thiazolo[3,2-b][1,2,4]triazolium cation depending on structural features using a combination of experimental and theoretical approaches. It was also demonstrated that the introduction of BAC into the exocyclic substituent of the thiazoline ring in [1,3]thiazolo[3,2-b][1,2,4]triazolium halogenides causes the appearance of the biological activity of these salts [20]. In the continuation of these studies and with the aim of explaining the impact of structural units of tested compounds on their biological effect, herein we focus on investigating the synthesis and antimicrobial evaluation of [1,3]thiazolo[3,2-b][1,2,4]triazolium salts, which contain BAC as an anion. For these purposes, we used proton-induced electrophilic heterocyclization with further anion-exchange reactions with appropriate acids. Perchlorate and hexabromotellurate anions served as BAC in our work. TeBr_6_^2−^ was chosen to extend our research on hybrid organic–inorganic materials with multifunctional properties, while ClO_4_^−^ was selected for its increased lipophilicity relative to halide anions, which tunes solubility and facilitates precipitation in our reactions. Also, recent data on the antimicrobial activity of the abovementioned scaffolds indicate the prospect of their biological relevance [20,21,23,24,25], despite the rather controversial issue of their toxicity [26,27].

## 2. Results and Discussion

### 2.1. Synthesis of [1,3]Thiazolo[3,2-b][1,2,4]triazol-7-ium Salts 2a–e

3-Methallylthio-1,2,4-triazoles **1** were obtained via the alkylation of corresponding 1,2,4-triazoles with methallyl chloride in the presence of a base under the described procedures [28,29]. Proton-induced electrophilic heterocyclization was carried out by the action of concentrated hydrobromic acid (40%) on a solution of heterocyclic precursor **1** in glacial acetic acid after 2 h of heating. It should be noted that the concentration and strength of the acid are critical factors for the electrophilic heterocyclization to proceed. Thus, dilute acids do not protonate the alkenyl moiety at a rate sufficient to produce appreciable amounts of the target products. Highly concentrated sulfuric acid forms a significant amount of tar, which dramatically reduces the yield, while the use of hydrochloric acid in combination with acetic acid (added to achieve acceptable solubility of the starting material) was ineffective—even after prolonged reflux, the yield remained low. Hydrobromic acid was chosen because it is stronger than hydrochloric acid, cost-effective, and much safer than other strong acids.

**Scheme 1:**
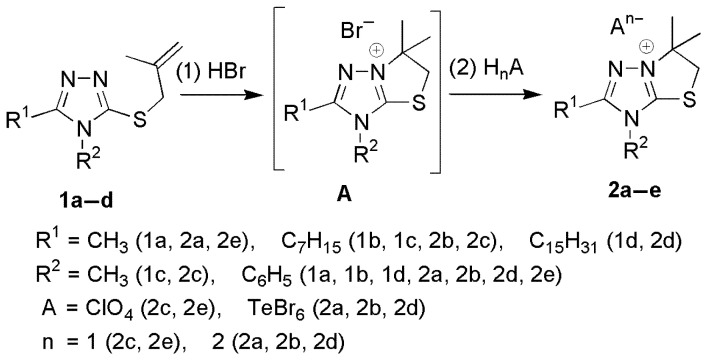
Synthetic approaches to [1,3]Thiazolo[3,2-b][1,2,4]triazol-7-ium salts **2a**–**e**.

The yielding bromides **A** were transformed without isolation into salts **2a**–**e** in an anion-exchange reaction via the action of the corresponding acid during the next step (Scheme 1).

The usage of a two-step procedure allows us to obtain hexabromotellurates **2a** and **2b** with higher yields in comparison with the direct action of hexabromotelluric acid described by us earlier [30], as well as ensuring the versatility of the approach of introducing anions of different natures for condensed salts **2a**–**e**.

### 2.2. Characterization of [1,3]Thiazolo[3,2-b][1,2,4]triazol-7-ium Salts 2a–e

**Figure 2:**
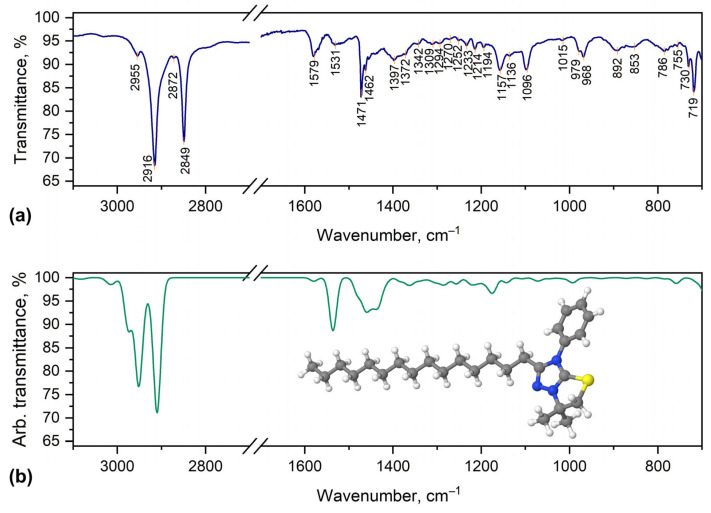
(**a**) Experimental FTIR spectra of compound **2d** and (**b**) theoretical IR spectra of cation 6,6-dimethyl-2-pentadecyl-3-phenyl-5,6-dihydro-3H-[1,3]Thiazolo[3,2-b][1,2,4]triazol-7-ium, calculated at the B3LYP/ma-def2-TZVP level of theory and scaled for 0.965. The optimized structure is shown as an inset.

**Figure 3:**
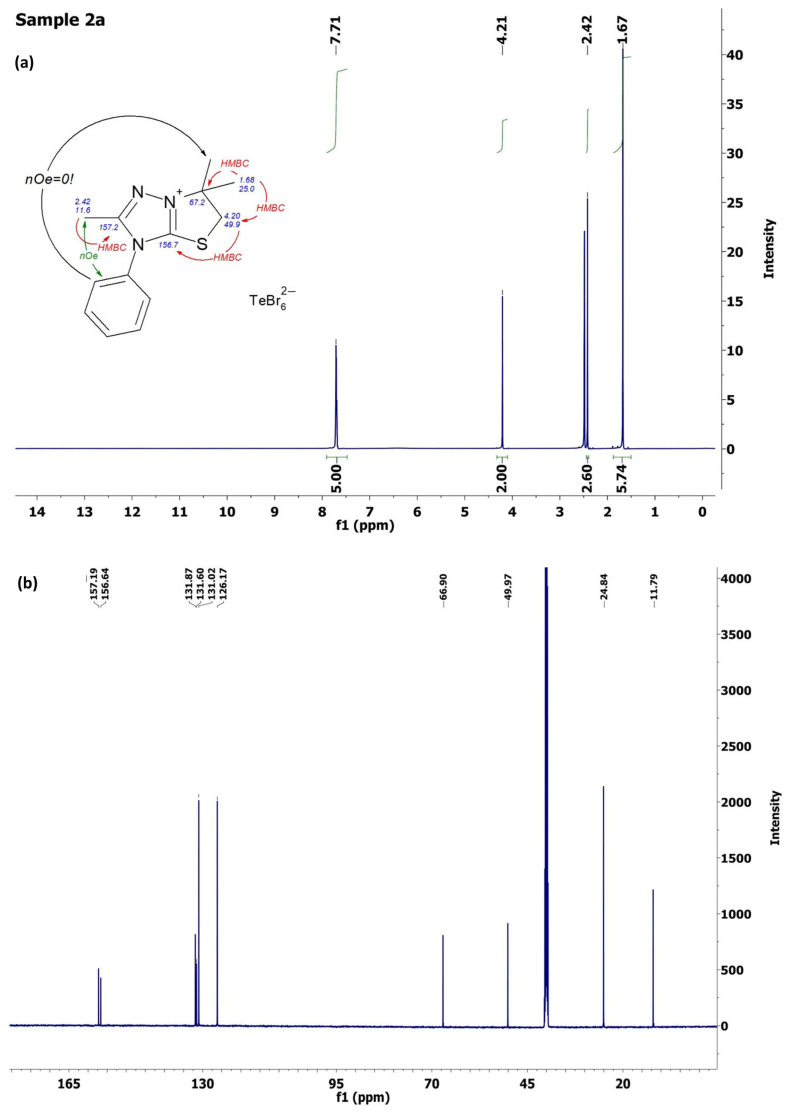
(**a**) 1H NMR and (**b**) 13C NMR spectra of compound **2a** in d6-DMSO.

The compositions of salts **2a**–**e** were determined by elemental analysis for C, H, N, and S, whose experimental values are close to the calculated ones and did not differ by more than 4%. The structures of the obtained [1,3]Thiazolo[3,2-b][1,2,4]triazol-7-ium salts **2a**–**e** were reliably confirmed by spectral data (Figure 2, Figure 3, Figures S1a,b and S2a,b); the purity was confirmed via liquid chromatography-mass spectrometry (LCMS) (Figures S3 and S4).

The FTIR spectra of salts **2a**–**e** contain all the peaks representing the expected vibrations. To assign the observed peaks to some specific vibrations, a comparison of the experimental and theoretical DFT-calculated spectra was made (Figure 2). The signals of the hexabromotellurate anion were not detected, as the corresponding vibrations were usually observed at 150–190 cm^–1^ [31]. Salts 2c and 2e contain an intensive band at ~1080 cm^–1^, which is caused by the perchlorate anion [32]. As a typical example, we will discuss the spectrum of **2a** in detail. The phenyl ring C–H stretching vibrations cause peaks at 3068 and 3060 cm^–1^. A peak at 3009 cm^–1^ can be assigned to C–H stretching vibrations of the SCH_2_ group. Alkyl substituents cause peaks at 2924, 2943, 2967, 2975, and 2982 cm^–1^, which correspond to asymmetric and symmetric C–H stretching. The lower values of wavenumber peaks in the region from 1593 to 717 cm^–1^ correspond to various C=C, C=N, and C–C stretching vibrations, as well as H–C–H, H–C–C, H–C–N, and H–C–S bending vibrations and H–C–C–H torsion vibrations.

The appearance in the ^1^H NMR spectra of compounds **2a**–**e** of a singlet peak at 1.67–1.78 ppm with an intensity of 6 (two methyl groups at C6 of the thiazolium ring at 1.67 ppm for compound **2a** in Figure 3a) and the absence of the signals of methallyl protons [30,31] unequivocally confirmed the closing of the 5-membered thiazoline cycle as a result of the electrophilic cyclization reaction.

Protons of the endocyclic S-CH_2_ group of salts **2a**–**e** are detected as a singlet at region 4.15–4.22 ppm, with an intensity of 2, for example, at 4.21 ppm for salt **2a** (Figure 3a). The signals of all protons of alkyl/phenyl were found and identified in the ^1^H NMR spectra of compounds **2a**–**e**. The spectral ^13^C NMR data (Figure 3b) also fully correlate with the structure assigned by us for salts **2a**–**e**. Thus, the signal of the thiazoline nodal C-6 and C-5 signals of the thiomethylene group of the [1,3]Thiazolo[3,2-b][1,2,4]triazol-7-ium cation in the abnormally weak region (at 66.1–67.1 ppm and at 48.7–50.0 ppm, accordingly) indicates the quaternization of the neighboring nitrogen atom, which occurs during the annellation of the thiazoline ring. The location of the carbon atoms’ signals from the triazole ring at 156.1–156.6 and 157.2–157.4 ppm also proves the formation of condensed [1,3]Thiazolo[3,2-b][1,2,4]triazol-7-ium salts **2a**–**e**. For salt **2a** (Figure 3b), the signals in the strong region at 11.8 ppm and 24.8 belong to methyl groups near C-2 in the triazole ring and near the C-6 nodal carbon, respectively, which fully confirms the assigned structure of compound **2a**.

Analysis of homo- and heteronuclear correlation data for compound **2a** (Figures S1a,b and S2a,b) provides reliable evidence for the formation of a thiazoline ring upon electrophilic cyclization.

All the salts, **2a**, **2b**, and **2d**, precipitated upon cooling the reaction mixture, while salts **2c** and **2e** were further recrystallized from the 1:3 water–ethanol mixture. The purity of the obtained target compounds **2a**–**e** was controlled by ^1^H NMR spectra, and for compounds **2a**,**b** additionally via LCMS (Figures S3 and S4).

### 2.3. Antifungal Activity

**Figure 4:**
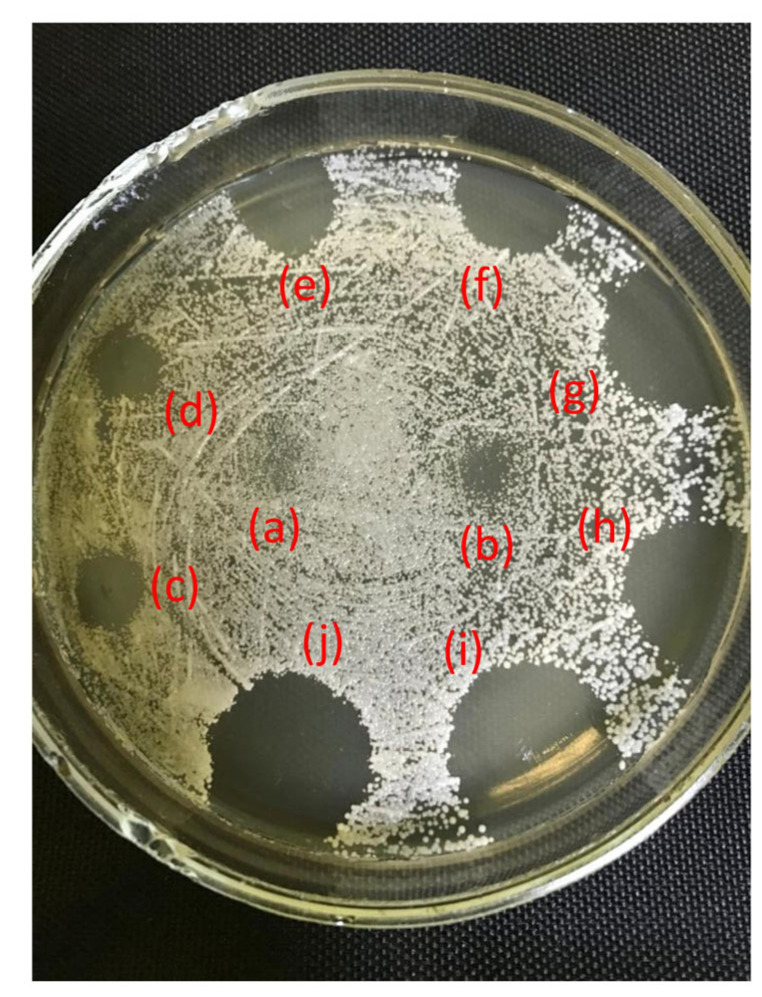
Preliminary evaluation of compound **2a** for antifungal action. Zones were produced into *Candida albicans* ATCC 885–653 lawns by compound **2a** applied onto and absorbed in Sabouraud agar in the periphery of the Petri dish as 20 µL drops at concentrations (**a**) 15.63, (**b**) 31.25, (**c**) 62.5, (**d**) 100, (**e**) 125, (**f**) 150, (**g**) 200, (**h**) 250, (**i**) 500, and (**j**) 750 µg/mL in advance of yeast suspension 10^6^ CFU/mL inoculation.

Only one compound out of five examined thiazolotriazoles, **2a**, was active against *Ascomycetes* fungi. As indicated by the inhibition zone size data (Table 1, Figure 4), compound **2a** exhibited higher antifungal activity against *Candida albicans* ATCC 885–653 than amphotericin B and nystatin, despite these antibiotics being present on testing disks at concentrations five and four times higher, respectively, than that of compound **2a**. However, the diameter of the inhibition zone for **2a** was smaller than those of ketoconazole, itraconazole, fluconazole, and clotrimazole, suggesting somewhat lower antifungal activity of the compound **2a** against *Candida albicans* ATCC 885–653 and BS3. However, the diameter of the inhibition zone for **2a** was smaller when compared to those of ketoconazole, itraconazole, fluconazole, and clotrimazole, suggesting somewhat lower antifungal activity of the compound **2a** against *Candida albicans* ATCC 885–653 and BS3. At the same time, these drugs produced smaller zones than compound **2a** in *Saccharomyces cerevisiae* BS3 lawns.

Thiazolotriazole **2a** suppressed *Candida albicans* ATCC 885–653, *Candida albicans* BS3, and *Saccharomyces cerevisiae* BS3 at a minimum inhibitory concentration (MIC) of 31.25 µg/mL and exhibited a fungicidal effect at the same minimum fungicidal concentration (MFC) of 125 µg/mL (Table 2). As is widely accepted, fluconazole is a fungistatic agent. In our study, it also exhibited only fungistatic action; however, it produced the largest zones in both *Candida albicans* strains and was selected as the standard for the microdilution test. The MICs of this antifungal drug against *Candida albicans* ATCC 885–653, *Candida albicans* BS3, and *Saccharomyces cerevisiae* BS3 were 31.25, 31.25, and 500 µg/mL, respectively (Table 2).

The data from disk diffusion and microdilution tests indicate that all fungal strains used in the test exhibited intermediate susceptibility to compound **2a**, according to the interpretive category of CLSI [33]. Additionally, the ratios MFC/MIC for the compound in all yeast strains did not exceed 4, suggesting the fungicidal properties of the compound.

Both strains of *C. albicans* were intermediate in susceptibility to fluconazole; however, the high MIC and 14 mm in diameter inhibition zone produced by the fluconazole disk in *Saccharomyces cerevisiae* BS3 lawn is evidence of this strain’s resistance to fluconazole.

Thus, from microdilution experiments, the compound **2a**, depending on concentrations, may have both fungicidal and fungistatic effects against strains of yeasts. The disk diffusion test revealed that **2a** exhibited a stronger antifungal effect at a dose of 20 µg/disk compared to nystatin and amphotericin B, even when these were applied at concentrations 4 and 5 times higher, respectively. Regardless of the same fungistatic doses for *Candida*, compound **2a** is superior compared to fluconazole because our compound possessed the fungicidal action lacking in fluconazole. Compound **2a** suppressed *Saccharomyces cerevisiae* BS3 at a dose about 18 times lower than fluconazole.

Our antifungal results show that only **2a** demonstrated strong antifungal activity. Reviewing the SAR data, we hypothesize that **2a**’s short, less bulky substituent optimizes interaction with fungal cell walls or ergosterol biosynthesis targets, facilitating better penetration and binding [34]. In contrast, the larger or more hydrophobic substituents in **2b**–**e** likely hinder membrane crossing or target engagement, resulting in weak or no antifungal effects [35].

Dozens of drugs for treating *Candida* infections are available on the market. However, most of them are able to cause side effects in patients (e.g., nephrotoxic effect of amphotericin B, hypersensitivity reactions, and cardiotoxic effects of echinocandins), and many have only fungistatic action (e.g., fluconazole). The resistance of pathogenic *Candida* to the polyene antibiotic amphotericin B was detected [36,37], and the resistance of these yeasts to azoles and echinocandins is present worldwide [38]. This makes the finding of new anticandidal remedies important.

Since the 1990s, there has been a growing number of *Saccharomyces cerevisiae* infections in humans. The symptoms are very similar to those in invasive candidiasis. A low susceptibility to amphotericin B and to azole derivatives was observed [39]. This makes the finding of new remedies very important. Compound **2a** (or its derivatives) could be further tested as a potential antifungal drug.

### 2.4. Antibacterial Activity

**Figure 5:**
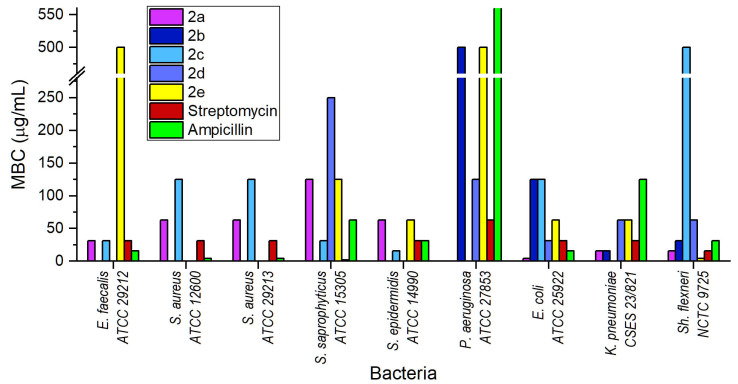
MBC values of thiazolotriazoles **2a**–**e** compared to streptomycin and ampicillin as standards.

**Figure 6:**
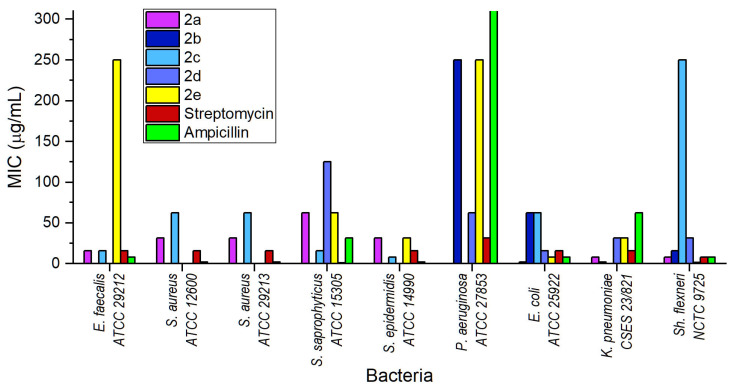
MIC values of thiazolotriazoles **2a**–**e** compared to streptomycin and ampicillin as standards.

Table 3 and Figure 5 and Figure 6 present the results of the antibacterial activity testing of the newly synthesized thiazolotriazoles on representative Gram-positive and Gram-negative bacteria, compared with the antibiotics streptomycin and ampicillin used as standards.

Particularly, thiazolotriazoles **2a** and **2c** showed good activity against all strains of *cocci* used in the experiment. The compound **2a** was able to have the lethal effects on *Staphylococcus epidermidis* ATCC 14,990, *S. saprophyticus* ATCC 15,305, *S. aureus* ATCC 29,213 or 12,600, and *Enterococcus faecalis* ATCC 29212 at the concentrations 62.5, 125, 62.5, and 31.25 µg/mL, while **2c** killed these bacteria at the concentrations 15.63, 31.25, 125, and 31.25 µg/mL, respectively. Bacteriostatic doses for **2a** and **2c** in suspensions of these strains were 31.25, 62.5, 31.25, and 15.63 µg/mL and 7.81, 15.63, 62.5, and 15.63, respectively.

Compound **2e** demonstrated good action against two strains of *Staphylococcus*, the *S. saprophyticus* ATCC 15305 with minimum bactericidal concentration (MBC) 125 and MIC 62.5 µg/mL and the *S. epidermidis* ATCC 14990 with MBC 62.5 and MIC 31.25 µg/mL. The **2e** had weak activity against *Enterococcus faecalis* ATCC 29212, with an MBC of 500 and MIC of 250 µg/mL, and no effect on both *S. aureus* strains. The compound **2d** was active only against *S. saprophyticus* ATCC 15,305, with an MBC of 250 µg/mL and MIC of 125 µg/mL, and compound **2b** showed no effect against Gram-positive *cocci* (Table 3, Figure 5 and Figure 6).

Similarly to those in streptomycin, the MBC/MIC ratios were less than or equal to 2 for compounds **2a**, **2c**, **2d**, and **2e** in all Gram-positive bacteria that were sensitive to them in our study. This finding proved the bactericidal action of our compounds against this group of microorganisms. According to the MBC/MIC ratios (less than or equal to 2), the antibiotic ampicillin demonstrated bactericidal action on all Gram-positive bacteria tested and was bacteriostatic against *S. epidermidis* ATCC 14,990 (MBC/MIC = 16).

The compounds **2a**–**e** possessed variable activity against Gram-negative bacteria also. The thiazolotriazoles **2a** and **2c** were inactive against *P. aeruginosa* ATCC 27853. High doses (500 µg/mL) of **2b** and **2e** compared to streptomycin (62.5 µg/mL) were necessary to kill the suspension of *P. aeruginosa* ATCC 27,853 cells. Concentrations higher than 125 µg/mL, a threshold for compounds to be regarded as active, as accepted in our study, are evidence of the bacterial resistance to these thiazolotriazoles. The MICs of the **2b** and **2e** compounds (250 µg/mL) were also higher than that of streptomycin (31.2 µg/mL). Compound **2d** exhibited satisfactory activity against *P. aeruginosa* ATCC 27853, with an MBC of 125 and an MIC of 62.5 µg/mL. Ampicillin was not active against *P. aeruginosa* ATCC 27,853 either, even at doses higher than 500 µg/mL.

All our compounds were active against enteric bacteria. Particularly, thiazolotriazoles **2a**, **2b**, **2c**, **2d**, and **2e** suppressed the growth of *Escherichia coli* ATCC 25,922 at MIC 1.95, 62.5, 62.5, 15.63, and 7.81 µg/mL and killed this enteric bacterium at MBC 3.9, 125, 125, 31.25, and 62.5 µg/mL, respectively. The MIC and MBC of **2a** for this bacterium were lower than such concentrations of streptomycin or ampicillin. The MIC of **2e** for *E. coli* ATCC 25,922 was also lower than in streptomycin and equal to that of ampicillin. The MBC of **2e** for this bacterium was higher than the MBC of these antibiotics. The **2d** compound has the same bactericidal and bacteriostatic concentrations as streptomycin has in microdilution tests against this microbe, and these are twice as high as in ampicillin. The compounds **2b** and **2c** both manifested the same bactericidal and bacteriostatic concentrations (125 and 62.5 µg/mL) against this strain, and they were higher compared to those of streptomycin (31.25 and 15.63 µg/mL) or ampicillin (15.63 and 7.81 µg/mL).

The thiazolotriazoles **2a** and **2b** were the best at killing and suppressing *Klebsiella pneumoniae* CSES 23/821 cells. The MBC and MIC of **2a** were 15.63 and 7.81 µg/mL, 2 and 8 times lower than those of streptomycin and ampicillin, respectively. Compound **2b** killed this capsule-forming bacterium at MBC 15.63 and totally suppressed its growth at MIC 1.95 µg/mL, at 2× and 8× or 8× and 32× times lower doses than those of streptomycin or ampicillin, respectively. The thiazolotriazoles **2d** and **2e** were active with the same MBC and MIC, 62.5 and 31.25 µg/mL, which are twice as low as those of ampicillin; however, this MBC and MIC are twice higher than the MBC and MIC of streptomycin. The compound **2c** was not active against *K. pneumoniae* CSES 23/821.

The thiazolotriazole **2a** demonstrated the same MBC and MIC as streptomycin against *Shigella flexneri* NCTC 9725: 15.63 and 7.81 µg/mL, respectively. The MBC of ampicillin for this bacterium was twice as high, and the MIC was the same as in **2a**. The MBC of **2b** was the same as in ampicillin; however, the MIC was twice as high. The compound **2c** killed or inhibited the microbe cells at high concentrations (MBC 500, MIC 250 µg/mL), suggesting the microbe is resistant to this thiazolotriazole. Both active doses of **2b** were twice as high as the MBC or MIC of streptomycin.

The MBC and MIC of **2d** were 4 times higher than those of streptomycin. The compound **2e** demonstrated the strongest action against *Shigella flexneri* NCTC 9725 with MBC 3.9 and MIC 1.95 µg/mL. These antimicrobial doses were several times lower than those of streptomycin and ampicillin (Table 3, Figure 5 and Figure 6).

Similarly to streptomycin and ampicillin, the MBC/MIC ratios were not higher than 4 for compounds **2a**, **2c**, and **2d** in all Gram-negative bacteria that were sensitive to them in our study. This proved the bactericidal action of these compounds and antibiotics on such bacteria. The compounds **2b** and **2e** had MBC/MIC ratios equal to 8 (more than 4) for *K. pneumoniae* CSES 23/821 and *E. coli* ATCC 25922, suggesting bacteriostatic action against these strains. The compounds **2b** and **2e**, with MBC/MIC ratios less than 4, demonstrated bactericidal effects on *Shigella flexneri* NCTC 9725 at low concentrations and against *P. aeruginosa* ATCC 27,853 at high concentrations. The latter bacterium was insensitive to **2a**, **2c**, and ampicillin and was the least sensitive bacterium among the strains tested against our compounds (Table 3).

According to MBC/MIC ratio calculations, all interactions between compounds **2a**–**e** and the tested bacteria were bactericidal, except of the action of **2b** on *K. pneumoniae* CSES 23/821 and **2e** on *E. coli* ATCC 25922.

In contrast, compound **2b**, with a longer heptyl chain and a phenyl group at N-3, entirely lost activity against Gram-positive bacteria but exhibited remarkable potency against certain Gram-negative pathogens, notably showing an MIC of 1.95 µg/mL on *K. pneumoniae*, surpassing both streptomycin and ampicillin. This shift indicates that increased hydrophobicity and steric bulk can hinder penetration of thick peptidoglycan layers while enhancing affinity for or uptake into Gram-negative outer membranes [40]. Compounds **2c** and **2e** displayed variable activity: **2c** retained good activity on Gram-positive strains similar to **2a**, whereas **2e** demonstrated exceptional potency specifically against *Shigella flexneri*, achieving an MIC of 1.95 µg/mL—significantly surpassing standard antibiotics. These findings suggest that specific chain lengths and subtle steric features finely tune antimicrobial spectrum and potency [41]. In contrast, compound **2d**, with the longest pentadecyl chain, showed limited activity overall, likely due to excessive lipophilicity and steric hindrance reducing effective bacterial penetration or target engagement [42]. Notably, MBC/MIC ratios ≤ 4 for compounds **2a**–**d** on sensitive strains confirmed their predominantly bactericidal action. These SAR insights highlight that moderate chain lengths with balanced hydrophobicity—as exemplified by **2a**—optimize broad-spectrum bactericidal properties, while longer, bulkier substituents favor selective Gram-negative activity, as seen with **2b**, but at the cost of Gram-positive efficacy [40,43]. Therefore, tailoring the length and nature of C-2 and N-3 substituents is critical for modulating antibacterial spectrum and potency in this series. Analysis of our antibacterial results revealed clear structure–activity relationships among the tested compounds. Compound **2a**, featuring a short methyl substituent at the C-2 position, consistently demonstrated the broadest and strongest bactericidal activity across both Gram-positive *cocci*, including *Enterococcus faecalis* and various *Staphylococcus* species, and certain Gram-negative strains like *Escherichia coli* and *Klebsiella pneumoniae*. This broad-spectrum potency suggests that a compact, moderately lipophilic structure favors the effective penetration of both Gram-positive cell walls and Gram-negative outer membranes, enabling efficient target binding [43,44].

Thus, compounds **2a**–**e**, depending on their structure and strains of microbes, demonstrated weaker, similar, or stronger activity compared to the action of streptomycin and ampicillin as standards against Gram-positive and Gram-negative bacteria. The current spread of antibiotic-resistant bacteria, particularly MRSA and their infections, poses the acute need for new drug development. Our compounds could possibly be of interest for such purposes.

### 2.5. Absorption, Distribution, Metabolism, Excretion, and Toxicity (ADMET) Analysis

The Absorption, Distribution, Metabolism, Excretion, and Toxicity (ADMET) studies were performed using ADMETlab 3.0 [45], an online tool, to assess the drug-like properties of the cations **2a**, **2b**, **2c**, and **2d** and to understand their potential as therapeutic agents. ADMET analysis is crucial in the early stages of drug development, as it helps predict a compound’s bioavailability, biological stability, and safety profile. By evaluating these key parameters, we aimed to identify compounds with the most favorable pharmacokinetic and pharmacodynamic properties, optimize their drug-like characteristics, and minimize potential risks, such as toxicity and poor solubility. The most important parameters are summarized, providing insights into their bioavailability and biological stability profiles, revealing both strengths and limitations for each compound presented in Table 4.

Thus, cation **2a** has moderate aqueous solubility (LogS) equal to −3.299 and a favorable lipophilicity (LogP) equal to 2.39, indicating potential for oral absorption, but its low human intestinal absorption (HIA-) and high P-gp inhibition reduce systemic availability. It also shows high plasma protein binding (80.21%), a limiting free drug concentration, and exhibits high metabolic instability with an ultra-short half-life (T_1/2_) of 0.411 h, raising concerns about rapid clearance. Toxicity predictions highlight a high risk of drug-induced liver injury (DILI) of 0.959, and a skin sensitization risk of 0.996.

Cation **2b**, with a higher molecular weight (330.2 g/mol) and LogP (4.256), suffers from poor solubility (LogS = −4.772) and very low intestinal absorption (HIA-), suggesting low oral bioavailability. It has high plasma protein binding (98.808%), low blood–brain barrier (BBB) penetration (0.247), and rapid metabolism (T_1/2_ = 0.231 h). Toxicity risks include DILI (0.844) and skin sensitization (0.994).

Cation **2c** shows moderate solubility (LogS = −3.525) and favorable intestinal absorption (HIA = 0.784), although its bioavailability is still low (F_50%_ = 0.995). It has a high plasma protein binding (94.149%) and low BBB penetration (0.102). It has a relatively stable profile (HLM stability = 0.402), though it still has a short T_1/2_ (0.274 h). Toxicity risks include DILI (0.771), skin sensitization (0.997), and respiratory toxicity (0.896).

Cation **2d** is the most lipophilic (LogP = 7.59), has very poor solubility (LogS = −6.399), and has extremely low intestinal absorption (HIA = 0.031), suggesting the least bioavailability. Its high plasma protein binding (100.551%) further reduces free drug availability. It is rapidly metabolized with a very short T_1/2_ (0.717 h), raising concerns about drug–drug interactions. Toxicity predictions highlight severe risks, including DILI (0.811), skin sensitization (0.999), and respiratory toxicity (0.971).

The comparative analysis of the bioavailability and biological stability of cations **2a**, **2b**, **2c**, and **2d** reveals significant differences in their ADMET profiles, which influence their therapeutic potential. Bioavailability, which reflects the extent and rate at which a compound reaches systemic circulation, varies widely among the compounds. Compound **2a**, with a molecular weight of 246.11, is the lightest and has a moderate LogP of 2.393, indicating a balance between hydrophilicity and lipophilicity that supports absorption. However, its solubility (LogS = −3.299) and intestinal absorption (HIA-) are limited. Compound **2b**, with a higher molecular weight of 330.2 and increased lipophilicity (LogP = 4.256), demonstrates even poorer solubility (LogS = −4.772), compounded by very low intestinal absorption (HIA-). Compound **2c** shows moderate solubility (LogS = −3.525) and the most favorable absorption profile (HIA+), but its bioavailability is still predicted to be below 50% (F_50%_ = 0.995). Compound **2d**, the heaviest at 442.33 g/mol, suffers from severe solubility issues (LogS = −6.399), extremely high lipophilicity (LogP = 7.59), and very low intestinal absorption (HIA-), making it the least bioavailable.

A closer examination of compounds **2a**, **2b**, and **2d** highlights how substituent length and lipophilicity critically influence both antibacterial activity and pharmacokinetic properties. Compound **2a**, featuring a shorter methyl group at C-2, demonstrated the broadest and most consistent bactericidal activity across both Gram-positive and certain Gram-negative strains. This favorable activity profile correlates with its moderate lipophilicity (LogP 2.39) and solubility (LogS −3.299), which together support sufficient membrane penetration while maintaining some degree of aqueous solubility. However, despite these promising in vitro effects, ADMET analysis revealed high plasma protein binding (80.2%), low predicted intestinal absorption, and rapid metabolic clearance, all of which may limit systemic exposure and therapeutic potential.

Compound **2b**, with its longer heptyl chain at C-2 and a phenyl group at N-3, displayed no activity against Gram-positive *cocci* but exhibited exceptional potency against specific Gram-negative bacteria such as *Klebsiella* pneumoniae. This selective activity is consistent with the compound’s higher lipophilicity (LogP 4.256), which may facilitate the penetration of Gram-negative outer membranes. Yet, this same hydrophobicity contributes to poor aqueous solubility (LogS −4.772), very low predicted intestinal absorption (HIA-), extremely high plasma protein binding (>98%), and a very short half-life, factors that collectively pose substantial challenges for systemic administration.

In contrast, compound **2d**, with the longest alkyl chain (pentadecyl) and phenyl group, showed limited antibacterial activity overall and possessed the most unfavorable ADMET profile of the series. Its extreme lipophilicity (LogP 7.59) and very poor solubility (LogS −6.399) translated into negligible predicted intestinal absorption, maximal plasma protein binding (~100%), and the highest predicted toxicity risks among the compounds studied. These findings suggest that extending the alkyl chain beyond an optimal threshold significantly impairs both bioavailability and safety without offering added antimicrobial benefits.

Overall, these comparisons underscore the importance of achieving a balance between hydrophobicity and solubility when designing thiazolotriazolium derivatives. Moderate chain lengths, as in **2a**, appear to best balance antimicrobial activity and drug-like properties, while excessive hydrophobicity, as seen with **2d**, compromises both efficacy and safety. Although **2b** demonstrates targeted potency against certain Gram-negative bacteria, addressing its ADMET liabilities through structural optimization or formulation strategies will be essential for further development. This analysis highlights the critical role of substituent size and lipophilicity in modulating both biological activity and pharmacokinetic behavior in this compound class.

In order to improve systemic exposure and reduce predicted toxicity risks, future optimization could focus on introducing small polar groups (e.g., hydroxyl, amine) at the ends of C-2 alkyl chains to increase solubility and reduce excessive lipophilicity, as well as limiting alkyl chain length to minimize plasma protein binding. Additionally, replacing or modifying the phenyl group at N-3 with less lipophilic or more hydrophilic substituents could decrease predicted hERG and DILI liabilities.

In terms of absorption parameters, all compounds show similar Caco-2 permeability values around -5, indicating moderate intestinal permeability. However, compounds **2a**, **2b**, and **2d** act as strong P-gp inhibitors, likely reducing their absorption through active efflux mechanisms, whereas compound **2c** is a weaker P-gp inhibitor and less prone to efflux. Plasma protein binding (PPB) significantly affects bioavailability, with **2d** showing the highest PPB (100.551%), leaving minimal free drug available for therapeutic action. Compound **2b** follows closely (98.808%), while **2c** (94.149%) and **2a** (80.217%) are less bound, providing a relatively freer drug in circulation. Blood–brain barrier (BBB) penetration is low for all compounds except **2a**, which shows a high probability of BBB crossing, suggesting potential central nervous system activity.

Biostability, determined by a compound’s resistance to metabolic degradation, is another major differentiator. Compounds **2a**, **2b**, and **2d** are all highly unstable in human liver microsomes, with HLM stability values close to 1, indicating rapid metabolism. Compound **2c**, with moderate HLM stability (0.402), stands out as the most stable. The half-life (T_1/2_) values further highlight the instability of these compounds, with **2b** (0.231 h) being the shortest, followed by **2c** (0.274 h), **2a** (0.411 h), and **2d** (0.717 h). These short half-lives, combined with moderate clearance rates (5–7 mL/min/kg), limit systemic retention for all compounds.

Toxicological profiles highlight significant risks across all compounds. DILI probabilities are high, with **2a** (0.959) being the most toxic. Skin sensitization is a consistent concern, with all compounds showing probabilities above 0.99, especially **2d** (0.999). Respiratory toxicity is also prominent, with compounds **2b**, **2c**, and **2d** exhibiting high probabilities. Cardiotoxicity risks, measured by hERG channel inhibition, are most notable in **2b** and **2d**, with probabilities of 0.431 and 0.697, respectively. While genotoxicity risks are moderate for **2a** and **2c**, they are minimal for **2d**, which has the lowest probability (0.002).

Overall, compound **2a** offers the best absorption potential due to its balanced physicochemical properties, but its bioavailability is limited by low intestinal absorption and high metabolic instability. Compound **2c** exhibits the highest biological stability, with moderate metabolic stability and a slightly longer half-life. However, its bioavailability is hindered by poor solubility and susceptibility to efflux. In contrast, compound **2d**, while being the most lipophilic, faces significant challenges due to poor solubility, high protein binding, rapid metabolism, and substantial toxicity risks, making it the least favorable of the group. To enhance their therapeutic potential, these compounds would require structural optimization or advanced formulation strategies to address their solubility, metabolism, and toxicity issues.

Based on the ADMET analysis, moving forward with the investigation of cations **2a**, **2b**, **2c**, and **2d** for pharmacological applications will require addressing several key challenges. Compound **2a**, while showing moderate solubility and good absorption potential, suffers from low intestinal absorption and high metabolic instability, which hinder its bioavailability. To improve its therapeutic potential, strategies such as prodrug design, formulation adjustments to enhance solubility, and the use of metabolic inhibitors could be explored. Compound **2b** presents significant challenges, with poor solubility, low absorption, and extensive metabolism, compounded by high toxicity risks. For this compound, formulation strategies to improve solubility and minimize toxicity risks, as well as the development of targeted delivery systems, are essential. Compound **2c**, although offering the best biostability and a moderate absorption profile, still faces limitations in bioavailability due to solubility and efflux issues. To optimize this compound, enhancing its solubility through formulation techniques or employing efflux pump inhibitors might improve systemic availability. Lastly, compound **2d**, while highly lipophilic, suffers from extremely low solubility, poor intestinal absorption, high plasma protein binding, and rapid metabolism, making it the least favorable. To move forward with **2d**, substantial modifications to improve its solubility and reduce toxicity, along with exploring advanced drug delivery systems, would be required. Overall, all four compounds show potential, but addressing their solubility, absorption, metabolism, and toxicity concerns through structural optimization and advanced formulation strategies will be key to improving their pharmacological viability.

### 2.6. Molecular Docking of Thiazolo[3,2-b][1,2,4]triazolium Cations 2a–2d

Molecular docking is an essential computational tool for understanding the interactions between ligands and target proteins, providing insights into structural compatibility and interaction dynamics. In this study, we selected UafA from *Staphylococcus saprophyticus* (PDB: 3IRZ) as a primary target for docking simulations involving a series of thiazolo[3,2-b][1,2,4]triazolium cations (**2a**, **2b**, **2c**, and **2d**) (Table 5). The rationale for choosing these proteins lies in their biological significance and their potential as therapeutic targets against bacterial pathogens.

UafA from *Staphylococcus saprophyticus* (PDB: 3IRZ) plays a key role in bacterial adhesion, a critical step in the colonization and infection processes of Gram-positive bacteria. This makes it a target for developing strategies to inhibit bacterial attachment and subsequent biofilm formation. The high-resolution crystal structure (1.70 Å) of UafA provides detailed atomic-level insights into its functional domain, enabling precise computational studies. Targeting UafA could contribute significantly to addressing the challenge of antibiotic-resistant Gram-positive bacteria by facilitating the development of novel adhesion inhibitors.

Targeting UafA provides an approach to combating bacterial infections by addressing essential processes in bacterial pathogenesis. This protein is integral to the structural and functional framework of Gram-positive bacteria, making it an ideal candidate for drug discovery efforts. The increasing prevalence of multidrug-resistant bacterial strains necessitates innovative approaches, and the exploration of this target supports the development of compounds with enhanced specificity and efficacy.

Our study began with docking simulations between the UafA protein from *Staphylococcus saprophyticus* and the thiazolo[3,2-b][1,2,4]triazolium cation **2a**. The results highlighted significant interactions between the ligand and specific amino acid residues within the UafA protein’s binding pocket. These interactions were characterized based on their energetic contributions and types. The ligand formed three non-bond interactions: a hydrophobic π–π T-shaped interaction with TRP511 and alkyl interactions with ILE685 and ILE554 residues. The cumulative binding affinity, calculated to be −6.0 kcal/mol, emphasized the favorable binding between the UafA protein and the investigated ligand.

Docking results for the interaction between the UafA protein and the 2-heptyl-3-phenyl-thiazolo[3,2-b][1,2,4]triazolium cation **2b** revealed several key interactions between the ligand and specific protein residues. π–σ interactions occurred between ILE478 and ALA735 and **2b**, while a π-sulfur interaction was observed between the sulfur atom of **2b** and PHE747. Alkyl interactions were formed with ILE478 and ALA735 residues, and a hydrophobic interaction occurred with ARG733. Additional interactions involved π–alkyl interactions between the ligand and various residues, including LEU734, ALA735, and LEU750. The calculated docking affinity of −6.8 kcal/mol suggests a strong binding between the UafA protein and the investigated ligand.

Heterocyclic cation **2c** formed a hydrophobic π–π T-shaped bond, engaging with the π-orbitals of TRP511 of the UafA protein, and a hydrophobic alkyl interaction with the residue ILE685. The 2-heptyl-3-methyl-thiazolo[3,2,-b][1,2,4]triazolium cation **2c** revealed an affinity of −5.3 kcal/mol, indicating a favorable interaction between the protein and the ligand.

The docking results between the UafA protein and the 2-pentadecyl-3-phenyl-thiazolo[3,2,-b][1,2,4]triazolium cation **2d** showed an electrostatic π–anion interaction with the negative charge of ASP425 through π-orbitals and hydrophobic alkyl interactions with ARG460, LEU557, and ILE685. Additionally, the ligand’s π-orbitals interacted with the alkyl group of ILE685, indicating a hydrophobic π–alkyl interaction. The affinity of this output was −5.1 kcal/mol.

Comparing the docking results for ligands **2a**, **2b**, **2c**, and **2d** interacting with the UafA protein from *Staphylococcus saprophyticus*, we observed some particularities. Cation **2b** exhibited the highest docking affinity (−6.8 kcal/mol) and a mix of π–σ, π–sulfur, alkyl, and π–alkyl interactions with the protein residues. Ligand **2a** followed with an affinity of −6.0 kcal/mol, predominantly forming alkyl interactions with hydrophobic residues, with one π–π T-shaped interaction. Cation **2c** scored a binding affinity of −5.3 kcal/mol, forming two non-covalent bonds—a π–π T-shaped interaction and an alkyl interaction. Finally, ligand **2d** (−5.1 kcal/mol) formed hydrophobic alkyl interactions with the protein residues, with an additional electrostatic π–anion interaction.

**Figure 7:**
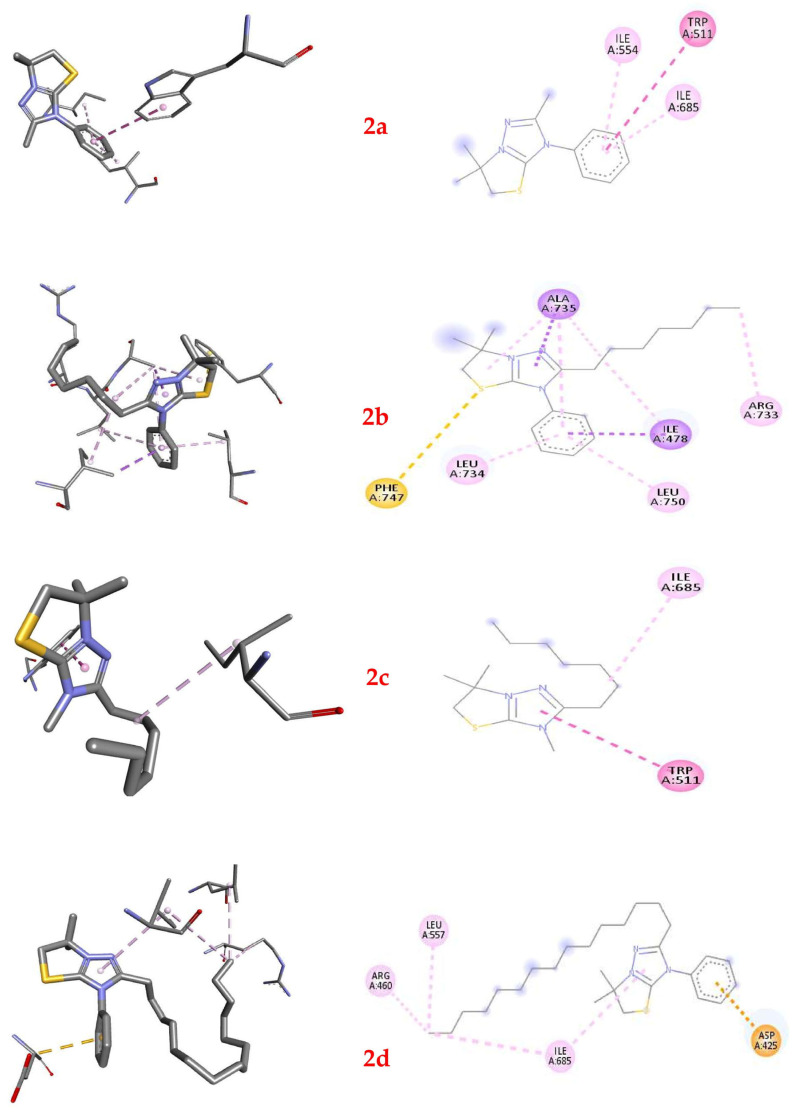
Docking of the best-generated conformers in 3D (**left**) and 2D (**right**) presentations for cations **2a**–**d** inside the binding pocket of UafA protein (PDB 3IRZ).

Analyzing the nature of substituents in positions C-2 and N-3 of the 1,2,4-triazole core, we found that the substitution pattern significantly influenced the type and strength of interactions between the ligands and the UafA protein. First of all, cations **2a**, **2c**, and **2d** interact in the same region of the protein, unlike cation **2b**, as is clearly seen from Figure 7. Cation with a pentadecyl substituent (**2d**) tended to form more extensive hydrophobic interactions, while ligands with heptyl substituents (**2b** and **2c**) exhibited a diverse range of interactions, including π–σ, π–sulfur, and π–π T-shaped interactions. Additionally, as seen from the example of cations **2a** and **2b**, the presence of a phenyl substituent could increase the binding properties, while the simultaneous effect of long-chain pentadecyl and phenyl substituents may negatively impact the binding affinity, as evidenced by ligand **2d**.

The nature of the substituents affected the overall binding affinity, with ligand **2b** exhibiting the highest affinity (−6.8 kcal/mol) among the studied ligands. These findings underscore the importance of carefully designing ligand structures to optimize their interactions with target proteins, thereby facilitating the development of novel therapeutics through structure-based drug design strategies.

To conclude, comparing the docking results of ligands **2a**, **2b**, **2c**, and **2d** interacting with the UafA protein from *Staphylococcus saprophyticus*, we observed notable differences in their interactions and binding affinities. Ligands **2a** and **2b** consistently demonstrated stronger binding affinities compared to ligands **2c** and **2d**. Ligands **2a**, **2b**, and **2d** exhibited interactions with multiple residues in the UafA binding pocket, while ligand **2c** formed fewer interactions overall. Ligands with shorter alkyl substituents at position C-2 (**2a** and **2b**) generally showed stronger binding affinities than ligand **2d**, which carries a longer pentadecyl chain. Additionally, the presence of the phenyl ring at position N-3 appears to increase binding affinity, as demonstrated by the higher affinity of ligand 2b compared to **2c**.

This pattern may be explained by the fact that longer substituents, such as the pentadecyl group in ligand **2d**, introduce steric hindrance that interferes with the optimal orientation of the ligand within the UafA binding pocket. This steric clash can disrupt favorable interactions with protein residues, resulting in weaker binding. Moreover, longer chains increase molecular flexibility, which may lead to less favorable conformations for effective binding. In contrast, shorter substituents allow the ligand to adopt a more constrained, stable conformation that better complements the binding site geometry. While hydrophobic interactions are important for ligand–protein binding, excessively long substituents may exceed the optimal surface area for hydrophobic contacts, undermining binding strength. Therefore, ligands with shorter chains tend to achieve a more balanced hydrophobic profile, leading to stronger binding affinities.

Ligand **2b**’s consistent superiority in both binding affinity and interaction diversity can be attributed to several factors. Its heptyl substituent strikes an optimal balance by providing sufficient hydrophobic interactions without excessive bulk. The moderate chain length allows favorable hydrophobic contact while avoiding steric hindrance. Additionally, ligand **2b** forms a diverse range of interactions, including π–σ, π–π T-shaped, and hydrophobic alkyl interactions. This diverse interaction profile enables engagement with multiple residues within the binding pocket, enhancing both binding affinity and specificity.

Overall, the length and structure of substituents critically influence ligand–protein interactions with UafA, with shorter and moderately hydrophobic chains generally favoring stronger binding affinities. Ligand **2b**’s optimal substituent length, diverse interactions, and specificity highlight its potential as a promising candidate for further study, emphasizing the importance of rational ligand design in the development of new antibacterial agents targeting UafA.

## 3. Materials and Methods

### 3.1. Chemicals and Instruments

All chemicals and solvents were sourced from Sfera Sim Ltd. (Lviv, Ukraine). Double-distilled water was used throughout all experiments. Nuclear Magnetic Resonance (NMR) spectra, including ^1^H NMR (400 MHz) and ^13^C NMR (100 MHz), were obtained with a Varian-Mercury 400 spectrometer. Fourier transform infrared (FTIR) spectra were collected using a Shimadzu IR Prestige-21 spectrometer (Shimadzu, Kyoto, Japan) in attenuated total reflection (ATR) mode, equipped with a MIRacle-A ATR accessory (PIKE Technologies, Inc., Madison, WI, USA) and a zinc selenide crystal. Mass spectra were measured on an Agilent 1100 LCMS SL instrument (Agilent, Santa Clara, CA, USA) with atmospheric pressure chemical ionization. Elemental analysis was conducted with the Elementar Vario MICRO cube analyzer (Elementar, Langenselbold, UK). Quantum chemical calculations were performed on a Dell PowerEdge server equipped with 256 GB of RAM and 40 Intel Xeon threads.

### 3.2. Synthesis of [1,3]Thiazolo[3,2-b][1,2,4]triazol-7-ium Salts 2a–e

A mixture of thioether **1** (3 mmol) in 4 mL of glacial acetic acid and 4 mL of concentrated hydrobromic acid (40%) was boiled in a water bath for 2 h. The cooled reaction mixture **A** was used to obtain the target salts **2a**–**e**.

3-Phenyl-2,6,6-trimethyl-5,6-dihydro-3*H*-[1,3]Thiazolo[3,2-b][1,2,4]triazol-7-ium hexabromotellurate **2a**: a bright orange–red solid was obtained from reaction mixture **A** by adding a solution of TeO_2_ (0.320 g, 2 mmol) in 7 mL of a 1:1 mixture of concentrated hydrobromic acid (40%) and glacial acetic acid with further heating at 1 h. The target salt **2a** was precipitated after the cooling, filtered, washed with 2 mL cooled glacial acetic acid and 5 mL diethyl ether, and dried in air at room temperature. Yield 81% (67% [30]). M. p. 277–279 °C (decompose). Anal. Calcd. (Found) for C_26_H_32_Br_6_N_6_S_2_Te, C: 28.40 (28.26), H: 2.93 (2.88), N: 7.64 (7.50), S: 5.83 (5.96). FTIR data (cm^−1^): 3068, 3060, 3009, 2982, 2975, 2967, 2943, 2924 (C–H); 1593, 1560, 1504, 1494, 1463, 1455, 1447, 1434, 1419 (C=N, C=C, C–N, C–C). ^1^H NMR (400 MHz, DMSO-d6) δ/ppm: 7.71 (s, 5H, C_6_H_5_), 4.21 (s, 2H, SCH_2_), 2.42 (s, 3H, CH_3_), and 1.67 (s, 6H, 2CH_3_). ^13^C NMR (100 MHz, DMSO-d6) ȏ/ppm: 157.2 (C=N), 156.6 (C=N^+^), 131.9 (C_6_H_5_), 131.6 (C_6_H_5_), 131.0 (C_6_H_5_), 126.2 (C_6_H_5_), 66.9 (C–N^+^), 50.0 (SCH_2_), 24.8 (2CH_3_), 11.8 (CH_3_). MS, m/z: 246 ${\left[M+H\right]}^{+}$. LC, RT 0.808 (100%).

2-Heptyl-6,6-dimethyl-3-phenyl-5,6-dihydro-3*H*-[1,3]thiazolo[3,2-*b*][1,2,4]triazol-7-ium hexabromotellurate **2b:** a bright orange-red solid was obtained from reaction mixture **A** by adding a solution of TeO_2_ (0.320 g, 2 mmol) in 7 mL of a 1:1 mixture of concentrated hydrobromic acid (40%) and glacial acetic acid with further heating at 1 h. The target salt **2b** was precipitated after the cooling, filtered, washed with 2 mL cooled glacial acetic acid and 5 mL diethyl ether, and dried in air at room temperature. Yield 85% (81% [30]). M. p. 158–160 °C. Anal. Calcd. (Found) for C_38_H_56_Br_6_N_6_S_2_Te, C: 35.99 (35.77), H: 4.45 (4.36), N: 6.63 (6.51), S: 5.06 (5.17). FTIR data (cm^−1^): 3059, 2984, 2944, 2925, 2862 (C–H); 1591, 1550, 1495, 1466, 1448, 1429 (C=N, C=C, C–N, C–C). ^1^H NMR (400 MHz, DMSO-d6) δ/ppm: 7.70 (s, 5H, C_6_H_5_), 4.19 (s, 2H, SCH_2_), 2.68 (t, *J* = 7.5 Hz, 2H, CH_2_), 1.68 (s, 6H, 2CH_3_), 1.55 (m, 2H, CH_2_), 1.14–1.24 (m, 8H, (CH_2_)_4_), and 0.84 (t, *J* = 6.5 Hz, 3H, CH_3_). ^13^C NMR (100 MHz, DMSO-d6) δ/ppm: 157.2 (C=N), 156.4 (C=N^+^), 131.5 (C_6_H_5_), 131.1 (C_6_H_5_), 130.6 (C_6_H_5_), 125.6 (C_6_H_5_), 66.5 (C–N^+^), 49.9 (SCH_2_), 30.8 (C_7_H_15_), 28.2 (C_7_H_15_), 27.6 (C_7_H_15_), 25.2 (C_7_H_15_), 25.0 (C_7_H_15_), 24.6 (2CH_3_), 21.8 (C_7_H_15_), 13.2 (CH_3_). MS, m/z: 330 ${\left[M+H\right]}^{+}$. LC, RT 1.315 (100%).

2-Heptyl-3,6,6-trimethyl-5,6-dihydro-3*H*-[1,3]thiazolo[3,2-*b*][1,2,4]triazol-7-ium perchlorate **2c:** white powder was obtained from reaction mixture **A** by adding 20 mL of water and further treating it with 15 mL of a 0.1 M solution of sodium perchlorate with the next precipitate filtration and recrystallization of the target salt **2c** with a water–ethanol 1:3 mixture. Yield 78%. M. p. 167–168 °C (decompose). Anal. Calcd. (Found) for C_14_H_26_ClN_3_O_4_S, C: 45.71 (45.52), H: 7.12 (7.00), N: 11.42 (11.50), S: 8.72 (8.88). FTIR data (cm^−1^): 2957, 2932, 2873, 2863 (C–H); 1575, 1525, 1503, 1464, 1453, 1408 (C=C, C=N, C-N), 1081 (ClO_4_^–^). ^1^H NMR (400 MHz, DMSO-d6) δ/ppm: 4.15 (s, 2H, SCH_2_), 3.64 (s, 3H, NCH_3_), 2.82 (t, *J* = 7.4 Hz, 2H, CH_2_), 1.78 (s, 6H, 2CH_3_), 1.67 (m, 2H, CH_2_), 1.28–1.39 (m, 8H, (CH_2_)_4_), and 0.89 (t, *J* = 6.5 Hz, 3H, CH_3_). ^13^C NMR (100 MHz, DMSO-d6) ȏ/ppm: 157.3 (C=N), 156.1 (C=N^+^), 66.2 (C–N^+^), 49.4 (SCH_2_), 32.8 (C_7_H_15_), 30.6 (C_7_H_15_), 30.1 (NCH_3_), 28.9 (C_7_H_15_), 25.2 (C_7_H_15_), 24.8 (C_7_H_15_), 24.6 (2CH_3_), 21.8 (C_7_H_15_), 13.1 (CH_3_).

2-Pentadecyl-6,6-dimethyl-3-phenyl-5,6-dihydro-3*H*-[1,3]thiazolo[3,2-*b*][1,2,4]triazol-7-ium hexabromotellurate **2d:** a bright orange–red solid was obtained from reaction mixture **A** by adding a solution of TeO_2_ (0.320 g, 2 mmol) in 7 mL of a 1:1 mixture of concentrated hydrobromic acid (40%) and glacial acetic acid with further heating at 1 h. The target salt **2d** was precipitated after cooling, filtered, washed with 2 mL of cooled glacial acetic acid and 5 mL diethyl ether, and dried in air at room temperature. Yield 81%. M.p. 122–124 °C. Anal. Calcd. (Found) for C_54_H_88_Br_6_N_6_S_2_Te, C: 43.46 (43.21), H: 5.94 (5.83), N: 5.63 (5.52), S: 4.30 (4.41). FTIR data (cm^−1^): 2955, 2916, 2872, 2849 (C–H); 1549, 1531, 1471, 1462 (C=N, C=C, C–N, C–C). ^1^H NMR (400 MHz, DMSO-d6) ȏ/ppm: 7.71 (s, 5H, C_6_H_5_), 4.18 (s, 2H, SCH_2_), 2.80 (t, *J* = 7.5 Hz, 2H, α-CH_2_), 1.74–1.66 (m, 2H, β-CH_2_), 1.64 (s, 6H, 2CH_3_), 1.52 (m, 2H, CH_2_), 1.22–1.34 (m, 22H, (CH_2_)_11_), and 0.85 (t, *J* = 6.5 Hz, 3H, CH_3_). ^13^C NMR (100 MHz, DMSO-d6) δ/ppm: 157.4 (C=N), 156.5 (C=N^+^), 131.6 (C_6_H_5_), 131.1 (C_6_H_5_), 130.6 (C_6_H_5_), 126.0 (C_6_H_5_), 66.1 (C–N^+^), 49.4 (SCH_2_), 32.4 (C_15_H_31_), 30.9 (C_15_H_31_), 29.0 (C_15_H_31_), 28.8 (C_15_H_31_), 28.6 (C_15_H_31_), 28.5 (C_15_H_31_), 28.0 (C_15_H_31_), 27.9 (C_15_H_31_), 25.2 (C_15_H_31_), 25.1 (C_15_H_31_), 25.0 (C_15_H_31_), 24.9 (C_15_H_31_), 24.6 (2CH_3_), 24.1 (C_15_H_31_), 21.9 (C_15_H_31_), 13.8 (CH_3_).

3-Phenyl-2,6,6-trimethyl-5,6-dihydro-3*H*-[1,3]Thiazolo[3,2-b][1,2,4]triazol-7-ium perchlorate **2e:** a white powder was obtained from reaction mixture **A** by adding 20 mL of water and further treating it with 15 mL of a 0.1 M solution of sodium perchlorate with the next precipitate filtration and recrystallization of the target salt **2e** with a water–ethanol 1:3 mixture. Yield 89%. M. p. 186–187 °C (decompose). Anal. Calcd. (Found) for C_13_H_16_ClN_3_O_4_S, C: 45.15 (45.32), H: 4.66 (4.75), N: 12.15 (12.27), S: 9.27 (9.19). FTIR data (cm^−1^): 3064, 3018, 2988, 2962 (C–H); 1595, 1568, 1517, 1501, 1470, 1456, 1428 (C=C, C=N, C-N); 1079 (ClO_4_^–^). ^1^H NMR (400 MHz, DMSO-d6) δ/ppm: 7.38–7.60 (m, 5H, C_6_H_5_), 4.22 (s, 2H, SCH_2_ ), 3.63 (s, 3H, CH_3_), and 1.68 (s, 6H, 2CH_3_). ^13^C NMR (100 MHz, DMSO-d6) ȏ/ppm: 157.2 (C=N), 156.6 (C=N^+^), 131.3 (C_6_H_5_), 125.5 (C_6_H_5_), 121.3 (C_6_H_5_), 120.1 (C_6_H_5_), 67.1 (C–N^+^), 48.7 (SCH_2_), 30.0 (NCH_3_), 24.8 (2CH_3_).

### 3.3. Microbial Strains

We used five Gram-positive *cocci Enterococcus faecalis* ATCC 29212, *Staphylococcus aureus* ATCC 12600, *Staphylococcus aureus* ATCC 29213, *Staphylococcus saprophyticus* ATCC 15305, *Staphylococcus epidermidis* ATCC 14990; four Gram-negative bacteria, *Escherichia coli* ATCC 25922, *Pseudomonas aeruginosa* ATCC 27853, *Klebsiella pneumoniae* CSES 23/821, *Shigella flexneri* NCTC 9725; and three strains of yeasts, *Candida albicans* ATCC 885–653, *Candida albicans* BS3, *Saccharomyces cerevisiae* BS3. The microorganisms were obtained earlier from the American Type Culture Collection, Rockville, MD, USA (ATCC), the Central Station of Sanitary and Epidemiology, Kyiv, Ukraine (CSES), and the National Collection of Type Cultures, Central Public Health Laboratory, London, UK (NCTC). Strains *C. albicans* BS3 and *S. cerevisiae* BS3 were isolated by the authors.

### 3.4. Antimicrobial Assays

#### 3.4.1. Antifungal Studies

To select the compounds with antifungal action, the thiazolotriazoles **2a**–**e** were preliminarily evaluated against *Ascomycetes*: strains of *Candida albicans* ATCC 885–653, *Candida albicans* BS3, and *Saccharomyces cerevisiae* BS3 in agar diffusion tests. All our compounds were applied as 20 µL drops of serial dilutions of 15.63, 31.25, 62.5, 100, 125, 150, 200, 250, 500, 750, and 1000 µg/mL onto preliminary dried at 50 °C for 15 min with Petri dish lids open on Sabouraud dextrose agar plates. These drops were absorbed into the medium in advance of 0.2 mL of yeast suspensions (10^6^ CFU/mL inoculations) using a glass swab. The dishes with *C. albicans* ATCC 885–653 and BS3 were incubated at 37 °C for 24 h, and plates with *Saccharomyces cerevisiae* BS3 were kept at 24 °C for 48 h. Following incubation, the inhibition zones were observed in the yeast lawns.

This enabled the identification of active compound **2a**, followed by its evaluation using the disk diffusion method in accordance with the CLSI procedure [33]. The disks, 6 mm in diameter, were cut from the Whatman No. 1 filter paper and sterilized within the glass Petri dishes in an oven at 160 °C for 45 min. Then, the disks were impregnated using a micropipette with DMSO solutions of salt **2a** to provide the final concentrations of the particular compound, 20 µg per disk. The 0.2 mL yeast suspensions (10^6^ CFU/mL according to the 0.5 McFarland standard) were inoculated as 20 µL drops onto the surface of the fresh Sabouraud dextrose agar plates and spread with a glass swab. Then, disks of our compounds were placed onto the surface of fresh seeded yeast lawns together with commercially supplied disks with the following content of the antifungal drugs: amphotericin B—100, nystatin—80, clotrimazole—10, fluconazole—20, itraconazole—10, and ketoconazole—20 µg. These drugs were used as standards for evaluation of inhibition zones in the lawns.

Compound **2a**, selected as active in the disk diffusion test, was further studied by the microdilution method with analogy to the techniques recommended by NCCLS and EUCAST [46]. Sabouraud dextrose broth (SDB) suspended in microtiter plates with 96 wells (Greiner Bio-One International GmbH, Kremsmünster, Austria) was utilized. Compound **2a** was dissolved in DMSO to the concentration of 10 mg/mL, and then 1 mL of this solution was diluted by 4 mL of SDB to provide the concentration of the compound **2a** of 2000 µg/mL. Then, 100 µL of this was poured into the 1st well of each of the well rows. Two-fold dilutions of compound **2a** were prepared by transferring 50 µL from the 1st to the 11th of 12 wells containing 50 µL of sterile SDB. The 12th well in each row was left without the addition of any compound and served as the growth control. The solutions in wells of each of the 8 rows were diluted 2-fold with 50 µL of fungal SDB inoculum 10^4^ cells/mL of each yeast strain to give the density 5 × 10^3^ cells/mL, and **2a** concentrations in a 100 mL final volume from 1000 µg/mL into the first well with a 2-fold decrease through 0.98 µg/mL in the 11th well. The *C. albicans* ATCC 885–653 or *C. albicans* BS3 were incubated at 37 °C for 24 h, and inoculations of *S. cerevisiae* BS3 were grown at 24 °C for 48 h. Drops of 5 µL of each of the dilutions were placed onto the centers of numbered squares of dried Sabouraud agar plates at 50 °C with open lids for 20 min. After absorption of the drops into the medium, the microbial cells were dispersed by a toothpick from the application site to the periphery of the square area and incubated at 24 °C or 37 °C for 2 days to distinguish the minimum fungicidal and inhibitive effect of the **2a** on yeasts, similarly to the method for differentiating bactericidal and bacteriostatic action of bacteriocins [46,47]. The lowest concentration at which no growth was observed after the absorbed drop spread was regarded as the minimum fungicidal concentration (MFC). The minimum inhibitory concentration (MIC) was that at which the spreading of drops resulted in less dense and smaller colonies, just before dilution with confluent colonies growth. The ratio MFC/MIC was estimated to verify if **2a** had fungicidal (MFC/MIC ≤ 4) or fungistatic (MFC/MIC ≥ 4) action.

The MFC/MIC ratio was estimated to verify if **2a** had fungicidal (MFC/MIC ≤ 4) or fungistatic (4 < MFC/MIC < 32) action, and if the strain could tolerate the compound (MFC/MIC ≥ 32) [48].

The fluconazole (“URiA PHARM”, Cherkassy, Ukraine), a derivative of triazole, was used as a standard. There were 3 replicates made of the experiment.

#### 3.4.2. Antibacterial Studies 

The obtained compounds **2a**–**e** were screened for their activity against Gram-negative and Gram-positive bacteria, according to the recommendations of CLSI 2012 [49]. Two-fold dilutions were prepared as described above (Section 3.4.1.) for all compounds **2a**–**e** and the two antibiotics used as standards: streptomycin and ampicillin. Mueller Hinton broth and Mueller Hinton agar (Merck KGaA, Darmstadt, Germany) were used in experiments with bacteria. The antibiotics were from PJSC “Kievmedpreparat” (Kyiv, Ukraine). The minimal fungicidal concentration (MBC) and minimal inhibitory concentrations (MIC) were estimated in the same way as MFC and MIC above, using the criteria of Hamon and Péron [47]. It was established earlier that antibacterial drugs should be regarded as cidal, static, or tolerant if ratios MBC/MIC are ≤ 4, between 4 and 32, or ≥32, respectively [50,51]. To estimate the **2a**–**e** compounds’ actions against bacteria, the MFC/MIC ratios were calculated for each compound–bacterium interaction. The experiment was repeated 3 times for each of the compounds and strains.

The in vitro antibacterial activity of synthetic compounds was compared to that of a reference antibiotic (streptomycin and ampicillin), and the results are supported by the results of a molecular docking investigation.

### 3.5. Software

#### 3.5.1. DFT Calculations

For an accurate assignment of experimental FTIR peaks to specific molecular vibrations, density functional theory (DFT) calculations at the B3LYP/ma-def2-TZVP level of theory [52,53,54,55] were performed (Figure 1—for salt **2d**). Geometry optimization and subsequent Hessian calculations were performed with the ORCA 5.0.3 package [56]. Analysis of the vibrational spectra was carried out with VibAnalysis [57].

#### 3.5.2. Molecular Docking Procedure

Molecular docking studies of cations **2a**–**d** were carried out using AutoDock Vina [58,59], an open-source molecular docking software. The docking simulations involved positioning the cations into the crystal structures of protein with PDB ID 3IRZ.

The methodology began with the optimization of the enzyme structures using BIOVIA Discovery Studio Visualizer 2021 [60]. This optimization process included defining the grid box around the active site of each protein to specify the docking search space, adding partial charges to the proteins to accurately reflect their electrostatic properties, and refining the protein structures to ensure the best possible representation of the biological targets.

For the preparation of the ligand structures, the cations **2a**–**d** were modeled using HyperChem 7 [61]. This involved optimizing their geometries to obtain the lowest energy conformations and ensuring that they were properly parameterized for docking studies.

Docking simulations were performed using AutoDock Vina’s advanced Genetic Algorithm method. For each docking experiment, we generated 10 distinct conformations of the ligands within the binding sites of the target proteins.

The docking input preparations, including setting up the receptor–ligand configurations and specifying the docking parameters, were managed within Discovery Studio software [62]. The software was also used for visualizing the docking results, analyzing binding affinities, and interpreting the interaction patterns between the cations and the protein targets.

## 4. Conclusions

The development of synthetic compounds that can combat drug-resistant pathogens is a key focus for scientific and medical research, particularly in countries facing resource constraints.

This research was focused on the accessible synthesis of [1,3]thiazolo[3,2-b][1,2,4]triazoles, a class of compounds combining two important 1,3-thiazole and 1,2,4-triazole scaffolds. Through electrophilic heterocyclization, several derivatives were synthesized and characterized using FTIR and NMR methods. These methods ensured the purity and structural integrity of the compounds, which were then evaluated for their antimicrobial potential. The results from the antimicrobial testing revealed that five of the synthesized compounds exhibited significant activity against a range of bacterial strains, including Gram-positive *Enterococcus faecalis* ATCC 29212, *Staphylococcus aureus* ATCC 12600, *Staphylococcus aureus* ATCC 29213, *Staphylococcus saprophyticus* ATCC 15305, *Staphylococcus epidermidis* ATCC 14990; and four Gram-negative bacteria: *Escherichia coli* ATCC 25922, *Pseudomonas aeruginosa* ATCC 27853, *Klebsiella pneumoniae* CSES 23/821, and *Shigella flexneri* NCTC 9725. The minimum inhibitory concentrations (MICs) of the compounds varied from 0.97 to 250 µg/mL, indicating a broad spectrum of activity. The minimum bactericidal concentrations (MBCs) were similarly promising, ranging from 1.95 to 500 µg/mL. Among these compounds, the 3-phenyl-2,6,6-trimethyl-5,6-dihydro-3H-[1,3]Thiazolo[3,2-b][1,2,4]triazol-7-ium hexabromotellurate was particularly notable, as it demonstrated strong activity against both bacterial and fungal strains. Its fungistatic activity against *Candida albicans* ATCC 885–653, *Candida albicans* BS3, and *Saccharomyces cerevisiae* BS3 was manifested at the dose of 31.25 µg/mL, the same as for fluconazole. The 3-phenyl-2,6,6-trimethyl-5,6-dihydro-3H-[1,3]Thiazolo[3,2-b][1,2,4]triazol-7-ium hexabromotellurate and the 2-heptyl derivative emerged as the most promising candidates for further development, with their activity profiles and molecular docking results supporting their potential for therapeutic use. These findings contribute to the ongoing search for new antimicrobial materials and offer hope for combating the growing issue of antimicrobial resistance. Future studies will focus on optimizing these compounds for enhanced bioactivity, understanding their mechanisms of action, and evaluating their safety and efficacy in vivo.

## Figures and Tables

**Table 1:** Inhibition zones caused by compound **2a** and antifungal drugs in lawns of yeasts.

Antifungal Compounds	6 mm Disk Content µg	Zone of Inhibition, mm *		
		*Candida albicans* ATCC 885–653	*Candida albicans* BS3	*Saccharomyces cerevisiae* BS3
Compound **2a**	20	20.3 ± 0.1	20.4 ± 0.2	20 ± 0.2
Amphotericin B	100	12.0 ± 0.2	11.5 ± 0.1	22 ± 0.3
Nystatin	80	13.2 ± 0.1	12.1 ± 0.2	21 ± 0.1
Clotrimazole	10	26.1 ± 0.2	24.5 ± 0.2	19.2 ± 0.2
Fluconazole	20	29.4 ± 0.1	29.5 ± 0.3	14.0 ± 0.3
Itraconazole	10	26.4 ± 0.3	25.5 ± 0.1	14.1 ± 0.2
Ketoconazole	20	29.1 ± 0.1	29.0 ± 0.1	13.7± 0.2

Note: * presented as mean diameter ± standard error.

**Table 2:** Antifungal action of fluconazole and thiazolotriazole **2a**.

Fungi	Thiazolotriazole 2a, µg/mL		Fluconazole, µg/mL	
	MFC	MIC	MFC	MIC
*Candida albicans* ATCC 885–653	125	31.25	-	31.25
*Candida albicans* BS3	125	31.25	-	31.25
*Saccharomyces cerevisiae* BS3	125	31.25	-	500

Note: MFC—minimum fungicidal concentration; MIC—minimum inhibitory (fungistatic) concentration; “-”—no antifungal action.

**Table 3:** Thiazolotriazoles **2a**–**e** compared to antibiotics streptomycin and ampicillin as minimum bactericidal concentrations (µg/mL) and minimum inhibitory (bacteriostatic) concentrations ( µg/mL).

Microorganism	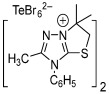 2a		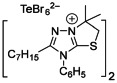 2b		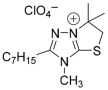 2c		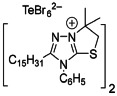 2d		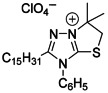 2e		Str		Amp	
	MBC	MIC	MBC	MIC	MBC	MIC	MBC	MIC	MBC	MIC	MBC	MIC	MBC	MIC
*Gram positive*														
*E. faecalis* ATCC 29212	31.25	15.63	-	-	31.25	15.63	-	-	500	250	31.25	15.63	15.63	7.81
*S. aureus* ATCC 12600	62.5	31.25	-	-	125	62.5	-	-	-	-	31.25	15.63	3.9	1.95
*S. aureus* ATCC 29213	62.5	31.25	-	-	125	62.5	-	-	-	-	31.25	15.63	3.9	1.95
*S. sapro-phyticus* ATCC 15305	125	62.5	-	-	31.25	15.63	250	125	125	62.5	1.95	0.97	62.5	31.25
*S. epidermidis* ATCC 14990	62.5	31.25	-	-	15.63	7.81	-	-	62.5	31.25	31.25	15.63	31.25	1.95
*Gram negative*														
*P. aeruginosa* ATCC 27853	-	-	500	250	-	-	125	62.5	500	250	62.5	31.25	>500	>500
*E. coli* ATCC 25922	3.9	1.95	125	62.5	125	62.5	31.25	15.63	62.5	7.81	31.25	15.63	15.63	7.81
*K. pneumoniae* CSES 23/821	15.63	7.81	15.63	1.95	-	-	62.5	31.25	62.5	31.25	31.25	15.63	125	62.5
*Sh. flexneri* NCTC 9725	15.63	7.81	31.25	15.6	500	250	62.5	31.25	3.9	1.95	15.63	7.81	31.25	7.81

Note: Str—streptomycin; Amp—ampicillin; MBC—minimum bactericidal concentrations; MIC—minimum inhibitory (bacteriostatic) concentrations; “-” compound was not active against the test culture at 500 µg/mL.

**Table 4:** The absorption, distribution, metabolism, excretion, and toxicity ADMET results for cations **2a**–**d**.

Parameter	Cation			
	2a	2b	2c	2d
Molecular Weight, g/mol	246.11	330.2	268.18	442.33
Topological Polar Surface Area (TPSA), Å^2^	21.7	21.7	21.7	21.7
Aqueous Solubility (LogS)	−3.299	−4.772	3.525	6.399
Lipophilicity (LogP)	2.393	4.256	3.409	7.59
Distribution (LogD)	2.584	3.704	3.079	4.844
Caco-2 Permeability	5.089	−5.032	5.069	−5.131
Intestinal Absorption (HIA)	HIA- (Low)	HIA- (Very Low)	HIA+ (Moderate)	HIA- (Very Low)
Plasma Protein Binding, %	80.217	98.808	94.149	100.551
BBB Penetration	High (0.885)	Low (0.247)	Low (0.102)	Very Low (0.029)
P-gp Inhibition	Strong Inhibitor (0.931)	Strong Inhibitor (0.99)	Weak Inhibitor (0.227)	Strong Inhibitor (0.867)
Human Liver Microsomal (HLM) Stability	Unstable (0.991)	Unstable (0.979)	Moderate Stability (0.402)	Unstable (0.914)
Half-life (T_1/2_), hours	0.411	0.231	0.274	0.717
Plasma Clearance, mL/min/kg	7.558	5.778	5.981	5.078
Bioavailability (F_50%_)	Low (0.868)	Low (0.976)	Low (0.995)	Very Low (0.997)
Drug-Induced Liver Injury (DILI)	High (0.959)	High (0.844)	High (0.771)	High (0.811)
Skin Sensitization	High (0.996)	High (0.994)	High (0.997)	High (0.999)
Respiratory Toxicity	Moderate (0.833)	High (0.895)	High (0.896)	High (0.971)
Carcinogenicity	Moderate (0.771)	Moderate (0.457)	Moderate (0.538)	Moderate (0.369)
Genotoxicity	Moderate (0.979)	Low (0.274)	Moderate (0.403)	Low (0.002)
Cardiotoxicity (hERG Inhibition)	Low (0.095)	Moderate (0.431)	Moderate (0.253)	High (0.697)

**Table 5:** Molecular docking results for cations **2a**–**d** and UafA target protein (*Staphylococcus saprophyticus*, PDB ID: 3IRZ).

Compound	Affinity, Kcal/Mol	Interacted Residue	Distance, Å
2a	−6.0	TRP511	5.24641
		ILE554	5.05906
		ILE685	5.36218
2b	−6.8	ILE478	3.73107
		ALA735	3.82162
		PHE747	5.46826
		ILE478	4.47169
		ALA735	5.09851
		ALA735	3.66844
		ARG733	4.64739
		LEU734	5.41669
		ALA735	5.47675
		LEU750	4.76871
2c	−5.3	TRP511	5.04091
		ILE685	4.7875
2d	−5.1	ASP425	3.45701
		ARG460	4.28296
		LEU557	4.83272
		ILE685	5.27912
		ILE685	4.88259

## Data Availability

The authors confirm that the data supporting the findings of this study are available within the article.

## Supplementary Materials

The following supporting information can be downloaded at https://www.mdpi.com/article/10.3390/ijms26146845/s1.
