# Supplementary material for: [1,3]Thiazolo[3,2-b][1,2,4]triazolium Salts as Effective Antimicrobial Agents: Synthesis, Biological Activity Evaluation, and Molecular Docking Studies
Source: Int J Mol Sci. 2025 Jul 16;26(14):6845. doi: 10.3390/ijms26146845 (PMC12294876; doi:10.3390/ijms26146845)
Supplement: Supplementary file 1 [file ijms-26-06845-s001.zip › ijms-3688746-supplementary.pdf]

Supplementary materials

[1,3]Thiazolo[3,2-b][1,2,4]triazolium Salts as Effective Antimicrobial Agents: Synthesis, Biological Activity Evaluation, and Molecular Docking Studies

Mykhailo Slivka [1], Boris Sharga [2], Daryna Pylypiv [3], Hanna Aleksyk [1], Nataliya Korol [1], Maksym Fizer [4], Olena I. Fedurcya [5], Oleksandr G. Pshenychnyi [5] and Ruslan Mariychuk [6,*]

[1] Educational Scientific Institute of Chemistry and Ecology, Uzhhorod National University, Fedyntsa str. 53/1, Uzhhorod, 88000, Ukraine; mikhailo.slivka@uzhnu.edu.ua (M.S.); hanna.hryhorka@uzhnu.edu.ua (H.A.); nataliya.korol@uzhnu.edu.ua (N.K.)

[2] Faculty of Biology, Uzhhorod National University, Voloshyna str. 32, Uzhhorod, 88000, Ukraine; boris.sharga@uzhnu.edu.ua (B.S.)

[3] Faculty of Medicine, Uzhhorod National University, Narodna Sq. 1, Uzhhorod 88000, Ukraine; darynapylypiv@gmail.com (D.P.)

[4] Department of Chemistry, University of Nevada, Reno, 1664 N. Virginia Street, Reno, Nevada 89557-0216, USA; mmfizer@gmail.com (M.F.)

[5] Transcarpathian Regional Center for Disease Control and Prevention of the Ministry of Health of Ukraine, Sobranetska str. 96, Uzhhorod 88000, Ukraine; fed.leno4ka@gmail.com (O.F.); agp0311cec@gmail.com (O.P.)

[6] Department of Ecology, Faculty of Humanity and Natural Sciences, University of Presov in Presov, 17th November str. 1, Presov 08001, Slovakia; ruslan.mariychuk@unipo.sk (R.M.)

* Correspondence: ruslan.mariychuk@unipo.sk (R.M.); Tel.: +421517570278

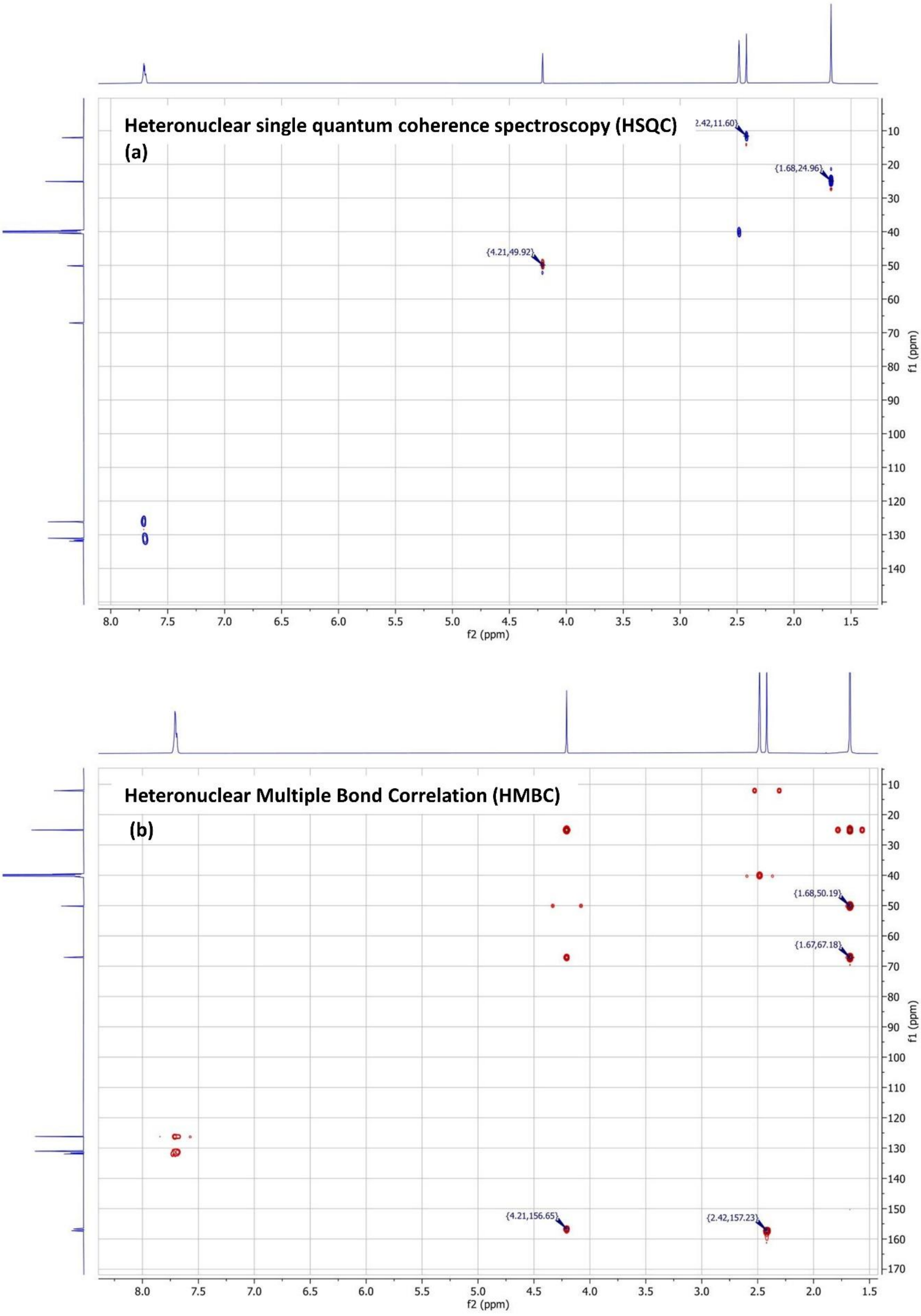

Figure S1. (a) Heteronuclear single quantum coherence spectroscopy and **(b)** Heteronuclear Multiple Bond Correlation (HMBC) for compound **2a**.

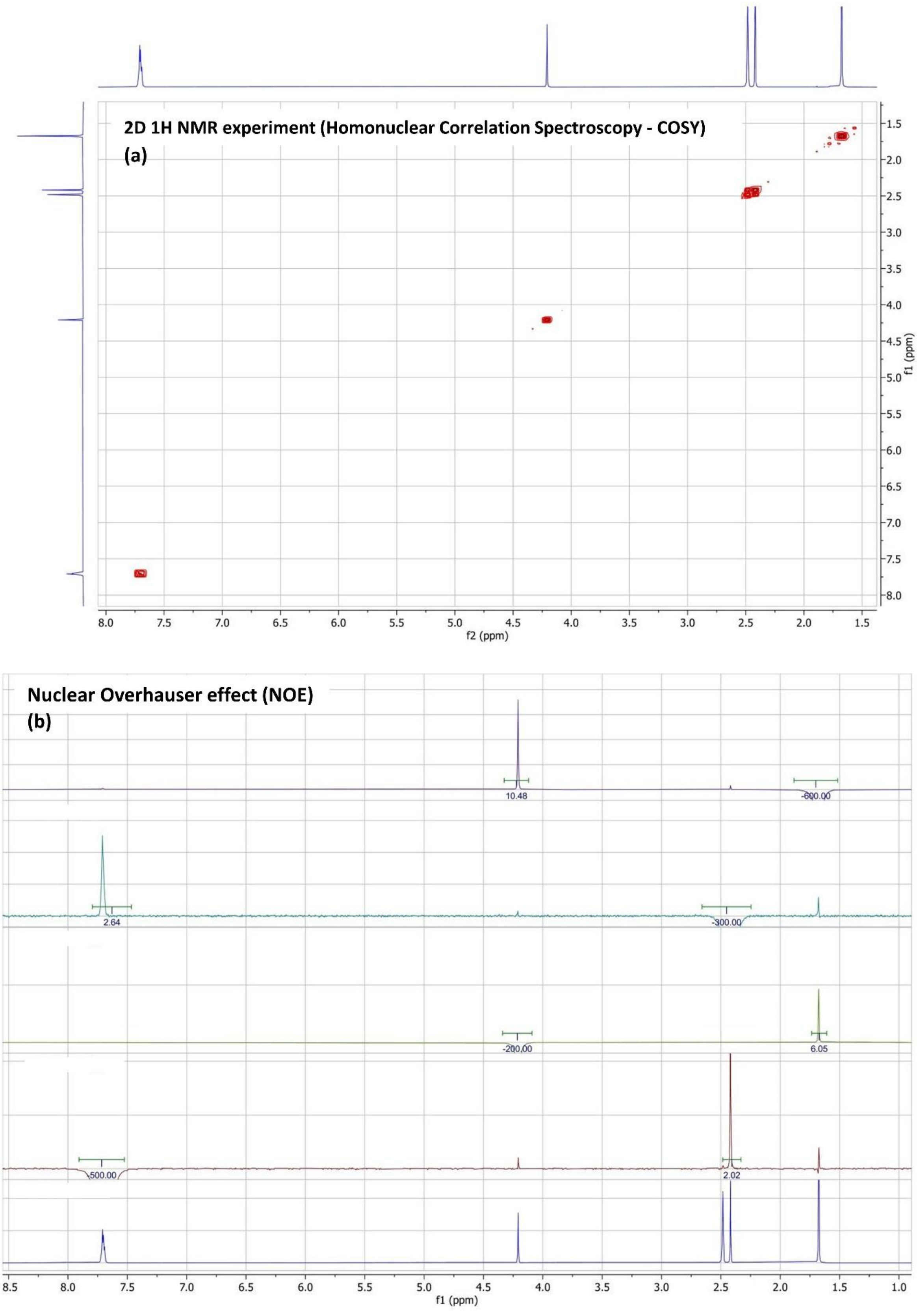

Figure S2. (a) Homonuclear Correlation Spectroscopy (COSY) and **(b)** Nuclear Overhauser effect (NOE) for compound **2a**.

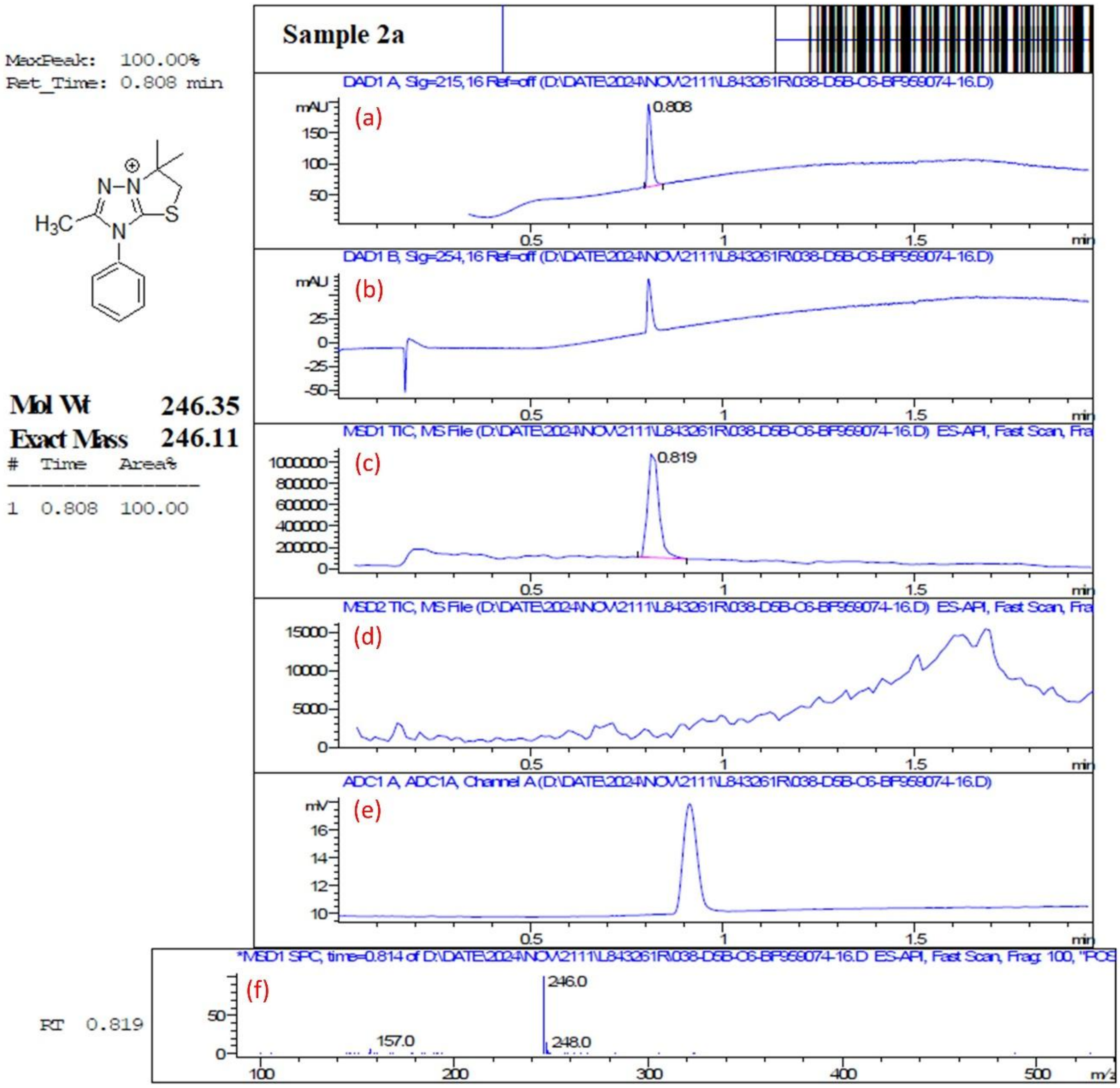

Figure S3. Chromatomass spectra of compound **2a** measured on an Agilent 1100 LCMSD SL instrument with chemical ionization (APCI): (a) LC chromatogram DAD1 A, Sig=215.16, Retention Time = 0.808; (b) LC chromatogram DAD1 B, Sig=254.16, Retention Time = 0.808; (c) TIC-MS file, MSD1, Retention Time = 0.819, ES-API, Fast Scan; (d) TIC-MS file, MSD2, Retention Time = 0.819, ES-API, Fast Scan; (e) – ACD1 A, Channel A, Retention Time = 0.819; (f) – MSD1 SPC, Retention Time = 0.819, ES-API, Fast Scan.

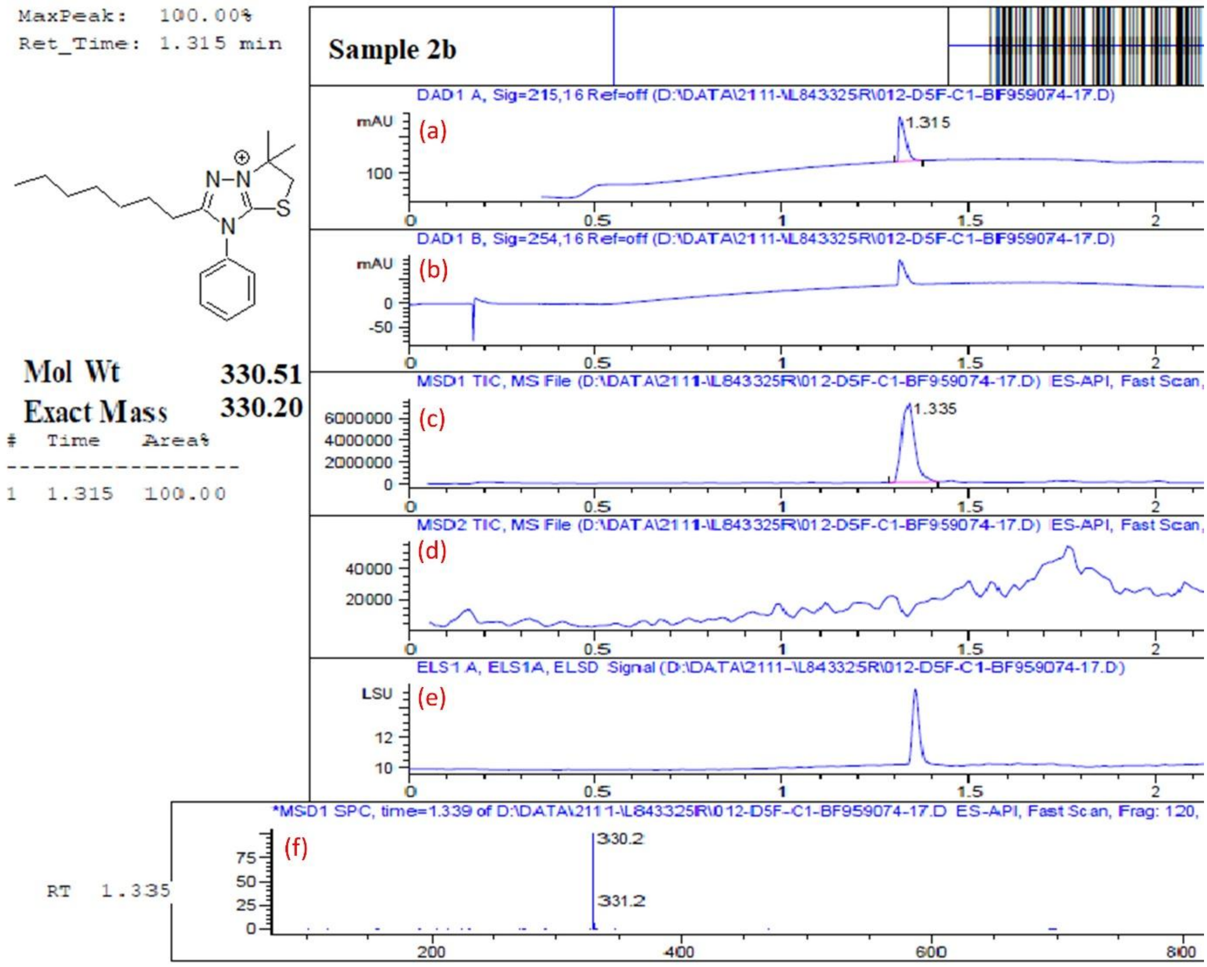

Figure S4. Chromatomass spectra of compound **2b** measured on an Agilent 1100 LCMSD SL instrument with chemical ionization (APCI): (a) LC chromatogram DAD1 A, Sig=215.16, Retention Time = 1.315; (b) – LC chromatogram DAD1 B, Sig=254.16, Retention Time = 1.315; (c) TIC-MS file, MSD1, Retention Time = 1.335, ES-API, Fast Scan; (d) TIC-MS file, MSD2, Retention Time = 1.335, ES-API, Fast Scan; (e) ACD1 A, Channel A, Retention Time = 1.335; (f) MSD1 SPC, Retention Time = 1.335, ES-API, Fast Scan.
